# A novel high-throughput screen identifies phenazine-1-carboxylic acid as an inhibitor of African swine fever virus replication in primary porcine alveolar macrophages

**DOI:** 10.1186/s13567-025-01467-2

**Published:** 2025-02-08

**Authors:** Jing Lan, Rui Luo, Di Liu, Changxing Qi, Xin Song, Zhanhao Lu, Ruojia Huang, Yuying Yang, Yuan Sun, Yonghui Zhang, Tao Wang, Hua-Ji Qiu

**Affiliations:** 1 State Key Laboratory for Animal Disease Control and Prevention, National African Swine Fever Para-Reference Laboratory, National High Containment Facilities for Animal Disease Control and Prevention, Chinese Academy of Agricultural Sciences, Harbin Veterinary Research Institute, Harbin, China; 2 College of Animal Science and Technology, Yangtze University, Jingzhou, China; 3 Hubei Key Laboratory of Natural Medicinal Chemistry and Resource Evaluation, Tongji Medical College, Huazhong University of Science and Technology, Wuhan, China

**Keywords:** African swine fever, African swine fever virus, dual-reporter virus, high-throughput screening, phenazine-1-carboxylic acid

## Abstract

African swine fever (ASF), caused by African swine fever virus (ASFV), has resulted in significant economic impacts on the global swine industry. Currently, there is no safe and effective commercial vaccine available for ASFV. Thus, the development of effective and readily available therapeutics for ASF is urgently needed. To conduct high-throughput screening (HTS) for anti-ASFV drugs, we initially developed a recombinant dual-reporter virus (rASFV-Gluc/EGFP) using the virulent strain ASFV HLJ/18 (ASFV-WT). The enhanced green fluorescent protein (EGFP)- and *Gaussia luciferase* (*Gluc*)-encoding genes were incorporated downstream of the ASFV *MGF300-4L* gene without disrupting viral genes. The growth kinetics, hemadsorption, and transmission electron microscopy analysis of rASFV-Gluc/EGFP in primary porcine alveolar macrophages (PAMs) revealed that rASFV-Gluc/EGFP exhibits similar biological characteristics to ASFV-WT. Furthermore, analysis of Gluc activities, fluorescence, and next-generation sequencing indicated that rASFV-Gluc/EGFP maintains good genetic stability after 20 consecutive passages in PAMs. Using the HTS platform established with rASFV-Gluc/EGFP, we screened and identified phenazine-1-carboxylic acid (PCA) as an effective inhibitor of ASFV replication from 246 small molecule compounds in PAMs. Importantly, PCA was found to reduce ASFV replication by as much as 100-fold at a concentration of 25 μM. Overall, this study suggests that rASFV-Gluc/EGFP is suitable for rapid screening of anti-ASFV drugs. Importantly, we showed that PCA has significant anti-ASFV activity in PAMs.

**Supplementary Information:**

The online version contains supplementary material available at 10.1186/s13567-025-01467-2.

## Introduction

African swine fever (ASF) is a highly contagious disease caused by African swine fever virus (ASFV), which primarily infects domestic pigs and wild boars [1]. It has caused significant economic losses to the global pig industry [2]. However, despite intensive efforts towards vaccine development, there is still a lack of safe and effective commercial vaccines against ASFV, except in Vietnam. Therefore, there is an urgent need for effective antiviral agents against ASFV, which could offer an alternative approach to combat this disease [3, 4]. To develop novel antiviral drugs, identifying drugs with substantial inhibitory effects among numerous potential candidates is imperative. Consequently, the establishment of efficient high-throughput screening (HTS) virological methods is paramount in evaluating a vast array of candidate drugs.

Owing to their high sensitivity and stability, fluorescent and luciferase proteins are utilized to generate reporter viruses [5, 6]. The choice of reporter genes is primarily dictated by the specific requirements of the study or application. Recombinant viruses expressing a fluorescent protein are typically used for in vitro studies to observe their cellular localization or identify infected cells. This property can also be used in HTS to calculate the infection efficiency of the virus by the ratio of fluorescence to the nucleus after staining the nucleus. Conversely, viruses expressing luciferase can be used for in vitro virus titration and imaging. The *Gaussia luciferase* (*Gluc*) gene is widely adopted as a reporter gene, enabling Gluc to be secreted from infected cells, with its activities measurable in the supernatants [7, 8]. A dual-reporter virus that co-expresses *Gluc* and enhanced green fluorescent protein (EGFP) allows both visualization of viral infection and rapid quantification of viral replication levels, representing an effective virological assay tool [6].

Several drugs have been reported to inhibit ASFV replication through distinct mechanisms. For example, rigid amphipathic fusion inhibitors and (S)-9-(3-hydroxy-2-phosphonylmethoxypropyl) adenine [(S)-HPMPA] inhibit viral DNA synthesis [9, 10]. Ailanthone targets prostaglandin E synthase 3 (p23) to inhibit the cochaperone cycle of heat shock protein 90 (HSP90), thereby hindering viral protein expression and suppressing ASFV replication [11]. Additionally, fluoroquinolones and genistein target ASFV topoisomerase II, which inhibits DNA synthesis [12, 13]. Bis-benzylisoquinoline alkaloids inhibit ASFV internalization and replication by alkalizing late endosomes/lysosomes [14]. Other compounds, such as triapine, cytarabine hydrochloride, dihydromyricetin, tetrandrine, berbamine, pentagastrin, cangrelor, fostamatinib, and polygalic acid, also exhibit inhibitory effects on ASFV replication in vitro [8, 15–18]. Despite these promising findings, most drugs or compounds are being evaluated in preclinical trials. Therefore, continued research is imperative to discover and validate novel compounds that can inhibit the replication of ASFV.

In this study, a dual-reporter virus (rASFV-Gluc/EGFP) expressing both *Gluc* and *EGFP* was constructed by integrating the *Gluc* and *EGFP* reporter genes downstream of the *MGF300-4L* gene through homologous recombination using ASFV HLJ/18 strain as the parental strain. The biological properties of rASFV-Gluc/EGFP were not significantly altered compared with those of ASFV-WT and that the virus maintained genetic stability. Using rASFV-Gluc/EGFP, we developed an HTS method. Among 246 small molecule compounds, we identified phenazine-1-carboxylic acid (PCA) as a potent inhibitor of ASFV replication in primary porcine alveolar macrophages (PAMs). Notably, PCA inhibited ASFV replication, achieving up to a 100-fold reduction at a concentration of 25 μM. PCA is a promising candidate for the development of novel anti-ASFV drugs.

## Materials and methods

### Cell, virus, and compound libraries

Human embryonic kidney 293 T (HEK293T) cells were cultured in Dulbecco’s modified Eagle’s medium (DMEM) (Gibco, 6123206) supplemented with 10% foetal bovine serum (FBS) (Gibco, A5669701) at 37 °C in a 5% CO_2_ incubator. PAMs were isolated from the lung lavage fluid of 28 day-old healthy specific-pathogen-free (SPF) piglets and maintained in RPMI 1640 medium with L-glutamine (Gibco, C11875500BT) supplemented with antibiotics (100 U/mL penicillin and 100 mg/mL streptomycin) and 10% FBS at 37 °C in a 5% CO_2_ incubator. The ASFV HLJ/18 strain (ASFV-WT) (GenBank accession number MK333180.1) was isolated as described previously [19]. The HEK293T cell-adapted ASFV (ASFV-P121) strain was generated as described previously [20]. Genistein (HY-14596) was purchased from Med Chem Express. Berbamine dihydrochloride (BAD) (T2920) and gamithromycin (GAM) (T3629) were purchased from Target Mol. Berbamine (BA) (s9141) was acquired from Selleck. The library containing 246 small molecule compounds is listed in Additional file 1.

### Generation of the dual-reporter ASFV

To generate a dual-reporter ASFV that co-expresses *EGFP* and *Gluc*, a transfer vector harbouring the *p72* promoter-controlled *Gluc* and *EGFP* genes was constructed. The *p72-EGFP* sequence was amplified from pOK12-EGFP-MGF300-4 L, while the *p72-Gluc* sequence was amplified from pOK12-p72-Gluc. Following amplification, the fragments were assembled in the order of the left arm, *p72-Gluc*, *p72-EGFP*, and right arm by overlapping polymerase chain reaction (PCR). The resulting fusion fragment was subsequently cloned and inserted into the pOK12 vector, generating pOK12-Gluc/EGFP. The primers used for sequence amplification are listed in Additional file 2.

PAMs were seeded in 6-well cell culture plates and incubated for 24 h at 37 °C. The PAMs were subsequently transfected with 2 μg of pOK12-Gluc/EGFP using X-tremeGENE HP (Roche, 6366546001) for 16 h and then infected with ASFV-WT at a multiplicity of infection (MOI) of 3. The recombinant viruses were harvested until EGFP expression, followed by multiple rounds of limiting dilution-based purification. The purified dual-reporter virus was validated by PCR and next-generation sequencing (NGS) as described below and designated rASFV-Gluc/EGFP.

### Luciferase assay

Gluc activities were measured using the *Gaussia luciferase* assay kit (Thermo Fisher Scientific, 16161) according to the manufacturer’s instructions. PAMs were infected with either ASFV-WT or rASFV-Gluc/EGFP. At 72 hours post-infection (hpi), the supernatants (30 μL) were collected and added to 96-well cell culture plates (black plate) (Biosharp, BS-MP-96B-CL). Next, the Gluc substrate (50 μL) was added to each well. The Gluc activities in the supernatants of each well were then measured using the EnVision HTS microplate reader (PerkinElmer).

### Hemadsorption (HAD) assay

PAMs were inoculated into 96-well cell culture plates (10^5^ cells/well), and ASFV-WT or rASFV-Gluc/EGFP was diluted to concentrations ranging from 10^–1^ to 10^–7^ in RPMI 1640 medium. The diluted virus was then added to the PAMs. At 4 days post-infection (dpi), 30 μL of 1% porcine red blood cells were added to each well. Hemadsorption was observed and expressed as 50% hemadsorption (HAD_50_) using the Reed & Muench method at 5 dpi [21].

### qPCR

The ASFV genomic DNA (gDNA) was extracted from the ASFV-infected PAMs using the nucleic acid extraction and purification kits (Tiangen, Y1814) according to the manufacturer’s protocols. The ASFV gDNA copies were then quantified by qPCR as described previously [22].

### Viral growth kinetics

Comparative replication kinetics between rASFV-Gluc/EGFP and ASFV-WT were performed in PAMs. PAMs were grown in 24-well cell culture plates and infected with either rASFV-Gluc/EGFP or ASFV-WT at an MOI of 5 for single-step growth curves or 0.01 for multistep growth curves. The samples were harvested at 2, 12, 24, 72, and 120 hpi. The viral titre was determined as described previously [20].

### Passaging and identification of the dual-reporter virus in PAMs

PAMs were infected with rASFV-Gluc/EGFP (passage 0, P0), and the supernatants were collected at 72 hpi. The resulting rASFV-Gluc/EGFP (P1) was used to infect PAMs to obtain P2, and the supernatants were also collected at 72 hpi. A total of 20 consecutive passages were conducted as described above. The EGFP fluorescence and Gluc activities of P1, P10, and P20 were examined to assess the genetic stability of the dual-reporter virus.

### NGS

To verify the genetic stability of the reporter virus, the ASFV gDNA of P0 and P20 was extracted from the infected PAMs using the QIAamp Blood Mini Kit (Qiagen, 51104), and the full-length sequence of the ASFV genome was determined by next-generation sequencing (NGS) as described previously [20].

### Transmission electron microscopy

To examine virus particle morphology, PAMs were infected with ASFV-WT or rASFV-Gluc/EGFP at an MOI of 5 and fixed with 2% glutaraldehyde in phosphate-buffered saline for 1 h at 18 hpi. The samples were dehydrated with acetone and embedded in epoxy according to a standard procedure. After polymerization, 80-nm-thick (ultrathin) sections were obtained and stained with uranyl acetate and lead citrate according to standard procedures [20]. The samples were analysed on an H-7650 (Hitachi, Tokyo, Japan) operated at 80 kV.

### Cytotoxicity assay

Cell cytotoxicity was measured using the CellTiter-Glo (CTG) luminescent cell viability assay kit (Promega, G7572) according to the manufacturer’s instructions. PAMs were cultured in medium in 96-well cell culture plates (10^5^ cells/well), and different concentrations of compounds were dissolved in dimethyl sulfoxide (DMSO) and added to the wells. The PAMs treated with the compounds were used as the experimental group, and a blank control group (RPMI 1640 medium + DMSO) and a negative control group (PAMs + RPMI 1640 medium + DMSO) were established. After the plates were incubated in the incubator for 48 h and equilibrated at room temperature for approximately 30 min, 100 μL of the CTG reagent was added to each well, and the contents of the PAMs were mixed for 2 min on an orbital shaker to induce cell lysis. Finally, the plates were incubated at room temperature for 10 min to stabilize the luminescent signals, and then 100 μL of the reaction solution was added to 96-well cell culture plates (black plate) (Biosharp, BS-MP-96B-CL) to measure the luminescent signal. The cell survival rate was calculated for each concentration according to the following formula: [(experimental group–blank group)/(negative control group–blank group)] × 100%.

### Western blotting

The PAMs infected with ASFV-WT or rASFV-Gluc/EGFP were harvested and lysed in ice-cold radioimmunoprecipitation assay (RIPA) lysis buffer (Sigma‒Aldrich, R0278) supplemented with a protease inhibitor cocktail (Roche, 4693116001). The cell lysates were boiled in SDS‒polyacrylamide gel electrophoresis (SDS‒PAGE) loading buffer (Solarbio, P1040) and resolved via SDS‒PAGE. The separated proteins were transferred onto polyvinylidene fluoride membranes, followed by blocking with 5% skim milk in Tris-buffered saline with 1% Tween 20 (TBST) for 1 h at room temperature. The membrane was incubated with anti-p72 and anti-A137R polyclonal antibodies [20] and a rabbit anti-*β*-tubulin (ABclonal, A12289) antibody preserved in our laboratory at 37 °C for 2 h, followed by washing three times with TBST. Finally, the membrane was incubated with IRDye 800CW goat anti-rabbit IgG (H + L) (LI-COR Biosciences, 926–32211) and goat anti-mouse IgG (H + L) (LI-COR Biosciences, 926–32212) secondary antibodies at 37 °C for 1 h and washed three times with TBST. The signals were detected by an Odyssey imaging system.

### HTS of anti-ASFV compounds

HTS was conducted in 96-well cell culture plates with a combination of rASFV-Gluc/EGFP and individual small-molecule compounds. The compound library, composed of 246 small-molecule compounds, was initially dissolved in DMSO to create a stock concentration of 10 mM. For primary screening, PAMs (10^5^ cells/well) were seeded in 96-well cell culture plates and incubated for approximately 12 h, followed by infection with rASFV-Gluc/EGFP (MOI = 0.2). Each compound at a concentration of 10 μM was added to the wells, with DMSO as a negative control. The luciferase activities of the compound-treated cells were quantified at 36 hpi via a Gluc assay system (Thermo Fisher Scientific, 16161) and an HTS microplate reader (PerkinElmer, ZY2020000385). The signal-to-background (S/B) ratio, coefficient of variation (CV), and Z-factor were calculated on the basis of Gluc activities to evaluate its stability and reproducibility in HTS. The calculation method was as follows: S/B = p/b; CV = *σ*_n_/*μ*_n_; Z factor =  (*σ* = standard deviation, p = positive control, n = negative control, b = blank control, *μ* = mean). The half-maximal cytotoxic concentration (CC_50_) was calculated by linear regression analysis of the dose‒response curves generated from the data. The half-maximal inhibitory concentration (IC_50_) was calculated by a nonlinear regression analysis of the dose‒response curves generated from the data. The selection index (SI) is a commonly used metric for assessing the therapeutic potential of a drug, with higher SI values indicative of a more potent drug in terms of antiviral efficacy. The calculation method is as follows: SI = CC_50_/IC_50_.

### Virus inactivation assay

rASFV-Gluc/EGFP (MOI = 1) and PCA (10 μM) were co-incubated at 37 °C for 2 h. After 100-fold dilution, the virus-PCA mixture was added to PAMs at a ratio of 1:1. Gluc and qPCR assays were conducted at 72 hpi to assess the impacts of PCA on rASFV-Gluc/EGFP replication.

### Time-of-addition assay

PAMs were seeded in 96-well plates and infected with rASFV-Gluc/EGFP (MOI = 0.2) for 1.5 h. PCA (10 μM) was administered at various time points before or after rASFV-Gluc/EGFP infection in PAMs, with a control group treated with DMSO established for comparison. At 24 hpi, the supernatants were collected for the Gluc assay.

### Statistical analysis

All statistical analyses were performed using Student’s *t* test or one-way ANOVA via the GraphPad Prism 8.0 software. The data are expressed as the mean ± standard deviation (SD). A *P* value of < 0.05 was considered statistically significant.

## Results

### Generation of rASFV-Gluc/EGFP

**Figure 1:**
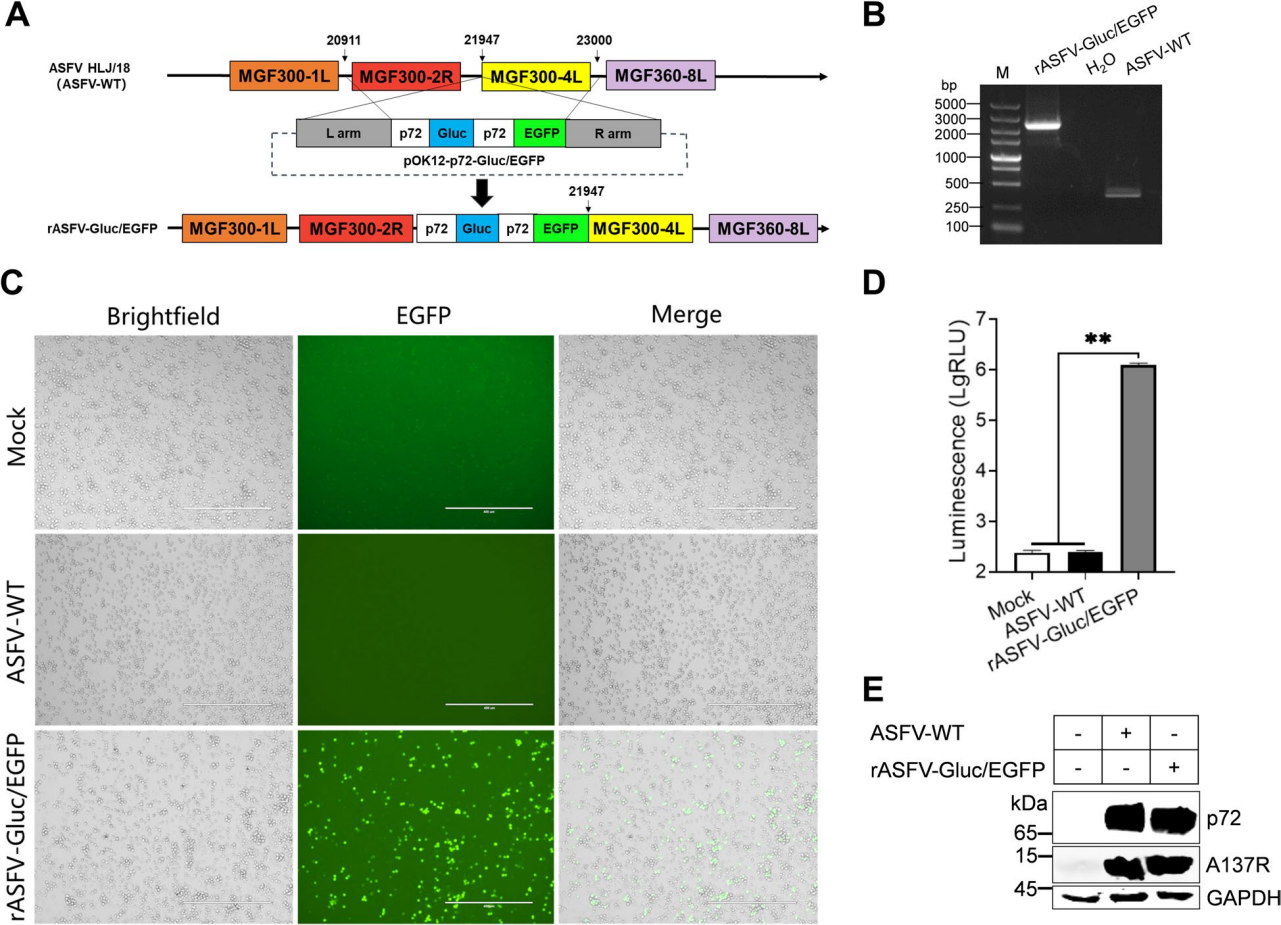
**Generation of a dual-reporter ASFV co-expressing Gluc and EGFP (rASFV-Gluc/EGFP). A** Schematic representation of the strategy for constructing the transfer vector pOK12-Gluc/EGFP and the dual-reporter virus rASFV-Gluc/EGFP. **B** PCR confirmation of rASFV-Gluc/EGFP purity. PCR amplification of genomic DNA derived from the ASFV-WT and rASFV-Gluc/EGFP genomes was performed using the primer pair D-JD-F/R, and the amplified products were subjected to gel electrophoresis. **C** Observation of EGFP expression. PAMs were infected with ASFV-WT or rASFV-Gluc/EGFP (MOI = 0.5), and EGFP expression was directly visualized via fluorescence microscopy at 72 hpi, with representative fluorescence images captured. Scale bars = 400 μm. **D** Gluc activity assay. PAMs were infected with either ASFV-WT or rASFV-Gluc/EGFP (MOI = 0.5), and the supernatants were collected at 72 hours post infection (hpi) to measure Gluc activities, which were expressed as relative light units (RLUs). **E** Western blotting analysis. The expression levels of the p72 and A137R proteins in the PAMs infected with rASFV-Gluc/EGFP or ASFV-WT were analysed by western blotting.

Previously, we engineered a reporter ASFV expressing *EGFP* by inserting the *EGFP* gene downstream of the *MGF300-4L* gene into the ASFV-WT genome [23]. Importantly, this approach did not hinder ASFV replication. Taking advantage of this, we employed a similar strategy to insert *Gluc* with *EGFP* genes downstream of the *MGF300-4L* gene. Specifically, the transfer vector pOK12-Gluc/EGFP was constructed by overlapping PCR (Figure 1A and Additional file 3). Following the transfection of HEK293T cells with pOK12-Gluc/EGFP and subsequent infection with ASFV-P121, Gluc activities were detected in the supernatants and, the EGFP expression was also detected from the ASFV-infected HEK293T cells (Additional file 3), indicating the successful construction of the transfer vector pOK12-Gluc/EGFP. The dual-reporter virus rASFV-Gluc/EGFP was generated via homologous recombination using pOK12-Gluc/EGFP (Additional file 3). The purification of rASFV-Gluc/EGFP was confirmed by PCR (Figure 1B). The NGS results confirmed the successful integration of the *Gluc* and *EGFP* genes (Additional file 4) and revealed no mutations or deletions at other loci within the ASFV genome. In parallel, the supernatants were collected to measure the Gluc activities, and the EGFP expression was observed using fluorescence microscopy. The results revealed that EGFP fluorescence was present in the PAMs infected with rASFV-Gluc/EGFP but was absent in the PAMs infected with ASFV-WT (Figure 1C). Similarly, Gluc activities were detectable in the supernatants of the rASFV-Gluc/EGFP-infected PAMs but not in those of the ASFV-WT-infected PAMs (Figure 1D). Furthermore, there was no significant difference in the expression levels of the p72 and A137R proteins in PAMs infected with rASFV-Gluc/EGFP or ASFV-WT, indicating that the insertion of the *Gluc* and *EGFP* genes does not affect the expression of viral proteins (Figure 1E). These results suggest the successful construction of rASFV-Gluc/EGFP, which is capable of expressing the *Gluc* and *EGFP* reporter genes.

### In vitro characterization of rASFV-Gluc/EGFP

**Figure 2:**
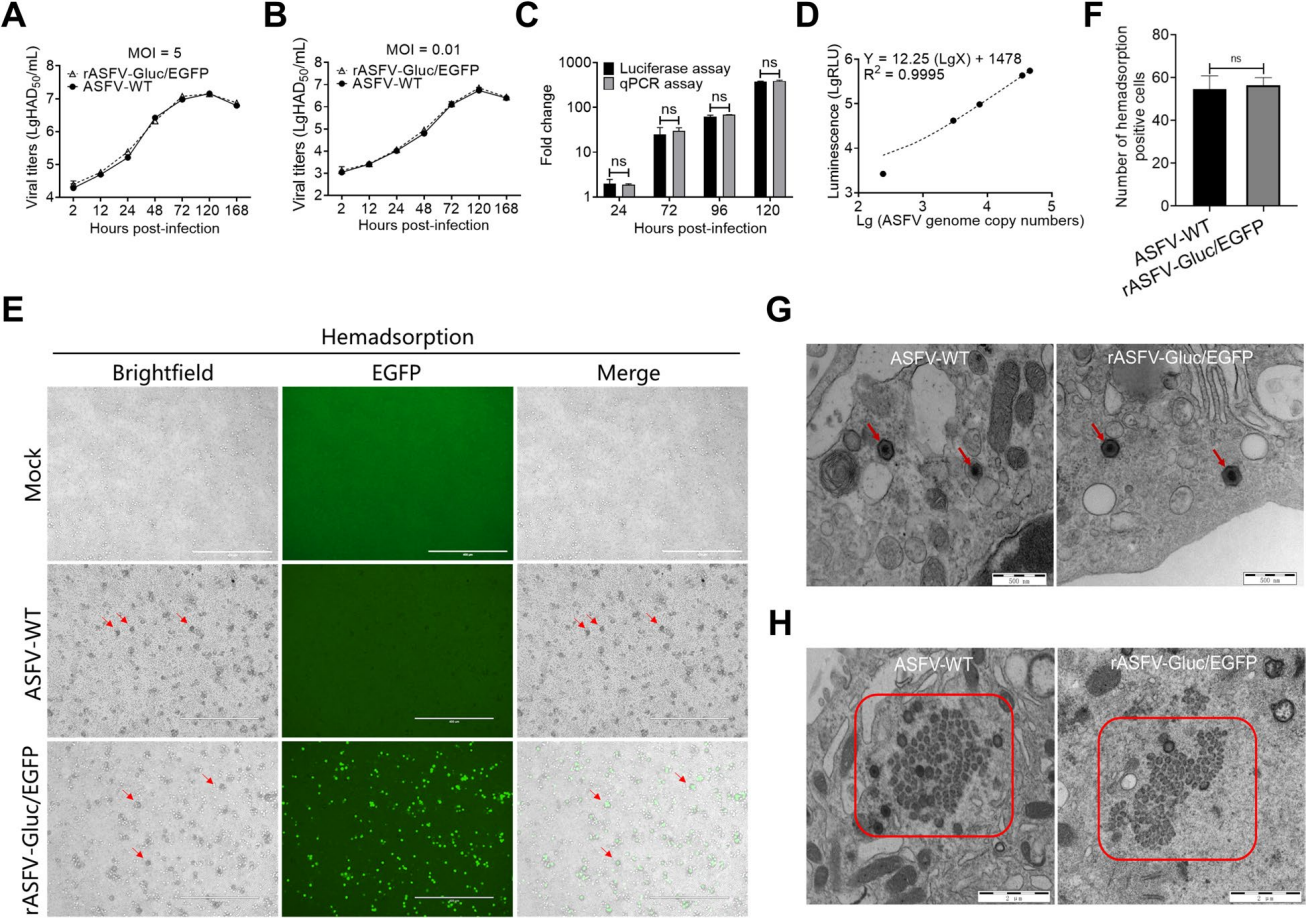
**In vitro characterization of rASFV-Gluc/EGFP. A** and **B** Growth kinetics of rASFV-Gluc/EGFP and ASFV-WT in PAMs. Single-step (MOI = 5) (**A**) and multi-step (MOI = 0.01) (**B**) growth curves were generated by measuring viral titres at various time points post-infection by the hemadsorption assay. **C** Correlation analysis between ASFV genome copies and Gluc activities. ASFV genome copies and Gluc activities were quantified in the PAMs infected with rASFV-Gluc/EGFP at 12, 24, 72, 96, and 120 hours post-infection (hpi), with the data normalized to 12 hpi for comparative analysis. **D** Linear regression analysis of rASFV-Gluc/EGFP genomic copies and Gluc activities. **E** and **F** Assessment of the hemadsorption of rASFV-Gluc/EGFP. Following infection with rASFV-Gluc/EGFP or ASFV-WT, fresh porcine red blood cells were added at 48 hpi, hemadsorption was observed (**E)**, and the number of hemadsorption-positive cells per field of view was counted using the ImageJ software (**F)**. Red arrows indicate hemadsorption. Scale bars = 400 μm. **G** and **H** Transmission electron microscopy (TEM) images of rASFV-Gluc/EGFP or ASFV-WT. PAMs were infected with rASFV-Gluc/EGFP or ASFV-WT, TEM was performed at 24 hpi, and representative images were selected. Red arrows point to viral particles (**G**). Scale bars = 500 nm. The red box shows the viral factory (**H**). Scale bars = 2 μm. ns, not significant.

To evaluate whether the incorporation of the *Gluc* and *EGFP* reporter genes affects viral replication, we examined the replication kinetics of rASFV-Gluc/EGFP in PAMs. The one-step (Figure 2A) and multistep (Figure 2B) growth curves revealed no significant difference in replication kinetics between rASFV-Gluc/EGFP and ASFV-WT. To determine whether rASFV-Gluc/EGFP can quantify the level of viral replication via Gluc, we compared the changes in Gluc activities and ASFV genome copies at different time points after infection with rASFV-Gluc/EGFP. Indeed, the PAMs infected with rASFV-Gluc/EGFP displayed no significant difference between the level of Gluc-related light units (RLUs) produced and the level of change in ASFV genome copies (Figure 2C), which were linearly correlated (Figure 2D).

A hemadsorption assay was subsequently performed to assess the effects of incorporating the *Gluc* and *EGFP* reporter genes on hemadsorption. Hemadsorption was observed in the PAMs infected with rASFV-Gluc/EGFP or ASFV-WT, but the difference was not significant (Figure 2E and F). These results suggest that the insertion of the *Gluc* and *EGFP* genes does not affect the hemadsorption properties of ASFV. Furthermore, via transmission electron microscopy, we detected no significant differences in the formation of viral factories or the morphology of viral particles in the PAMs infected with rASFV-Gluc/EGFP or ASFV-WT (Figure 2G and H). These findings indicate that the insertion of the *Gluc* and *EGFP* genes does not impact the viral particle morphology of ASFV.

### The genetic stability of rASFV-Gluc/EGFP

**Figure 3:**
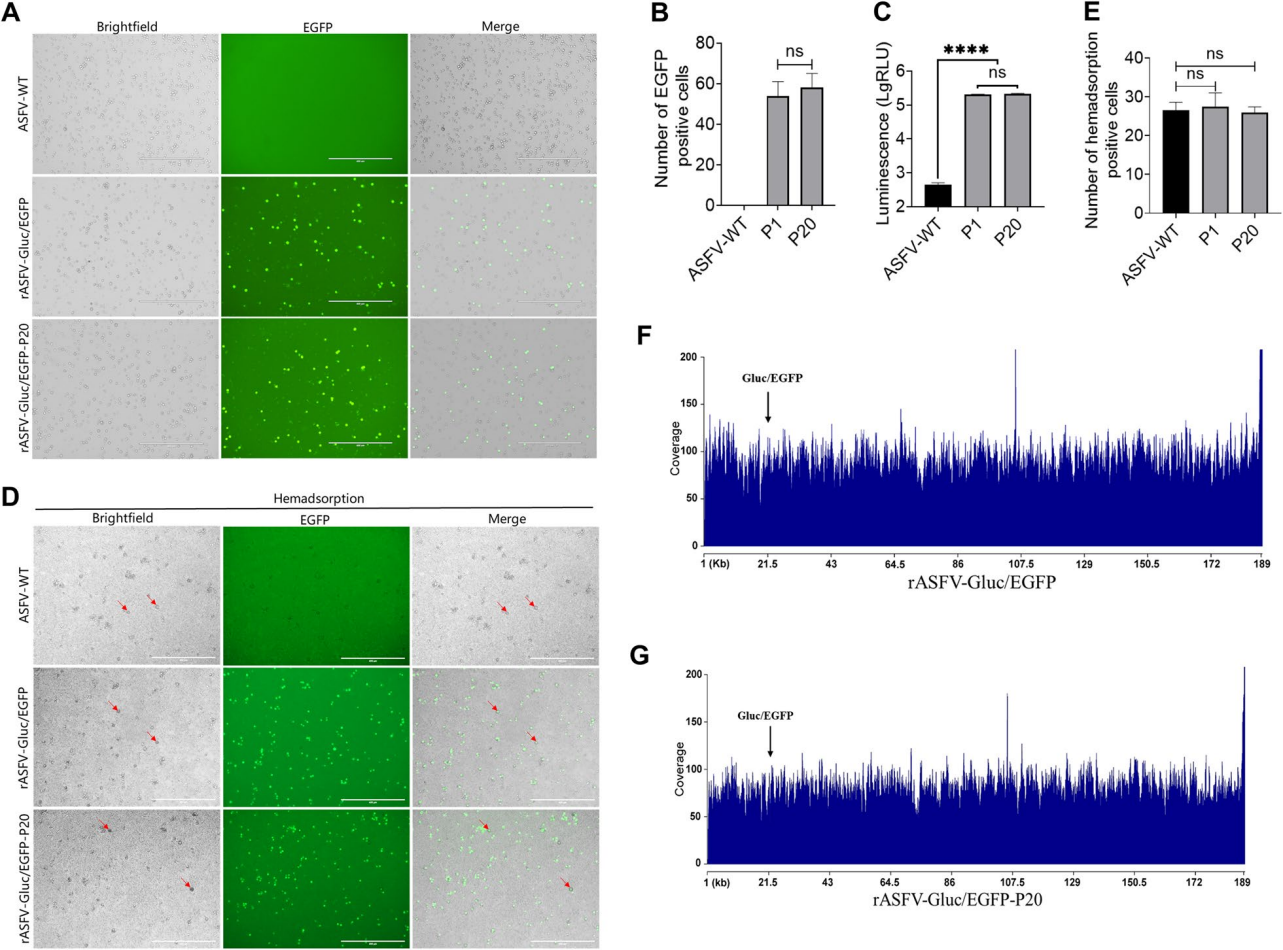
**ASFV-Gluc/EGFP is genetically stable. A** and **B** Stability of EGFP expression. rASFV-Gluc/EGFP was subjected to 20 consecutive passages in PAMs, and EGFP expression was observed by fluorescence microscopy at 72 hours post-infection (hpi) for P1 and P20, with representative fluorescence images captured (**A**) and the number of EGFP-positive cells per field of view counted using the ImageJ software (**B**). Scale bars = 400 μm. **C** Stability of the Gluc activities. rASFV-Gluc/EGFP was subjected to 20 consecutive passages in PAMs, and Gluc activities were assayed at P1 and P20 at 72 hpi. **D** and **E** Stability of the hemadsorpting capability. rASFV-Gluc/EGFP was subjected to 20 consecutive passages in PAMs, fresh porcine red blood cells were added to P1 and P20 at 72 hpi, and hemadsorption was observed (**D**). The number of hemadsorption-positive cells per field of view was measured using the ImageJ software (**E**). Red arrows indicate hemadsorption. Scale bars = 400 μm. **F** and **G** NGS analysis of the complete genome of rASFV-Gluc/EGFP. A complete genome coverage plot using the NGS reads of rASFV-Gluc/EGFP (**F**) and rASFV-Gluc/EGFP-P20 (**G**) was generated by mappingvia mapping against the ASFV-WT genome. The read coverage was analysed using SAMtools (version 1.3.1) and visualized using RStudio software (version 3.6.1). ****P < 0.0001; ns, not significant.

To evaluate the genetic stability of rASFV-Gluc/EGFP, the virus was passaged 20 times in PAMs (Additional file 5). Throughout the passage process, the EGFP expression and Gluc activities in the supernatants collected from P1, P10, and P20 were monitored (Figures 3A and B and Additional file 5). The results revealed that P10 and P20 were still able to express *EGFP*, and there was no significant difference in Gluc activities between P1 and P20 (Figure 3C). Moreover, the hemadsorpting characteristics of rASFV-Gluc/EGFP remained unchanged during the passage process (Figure 3D and E and Additional file 5). NGS revealed no undesired deletions, mutations, or recombinations in the reporter genes or viral genes between P0 and P20 (Figures 3F and G and Additional file 4). These findings demonstrate that rASFV-Gluc/EGFP is genetically stable throughout 20 consecutive passages in PAMs.

### Establishment of an HTS using rASFV-Gluc/EGFP

**Figure 4:**
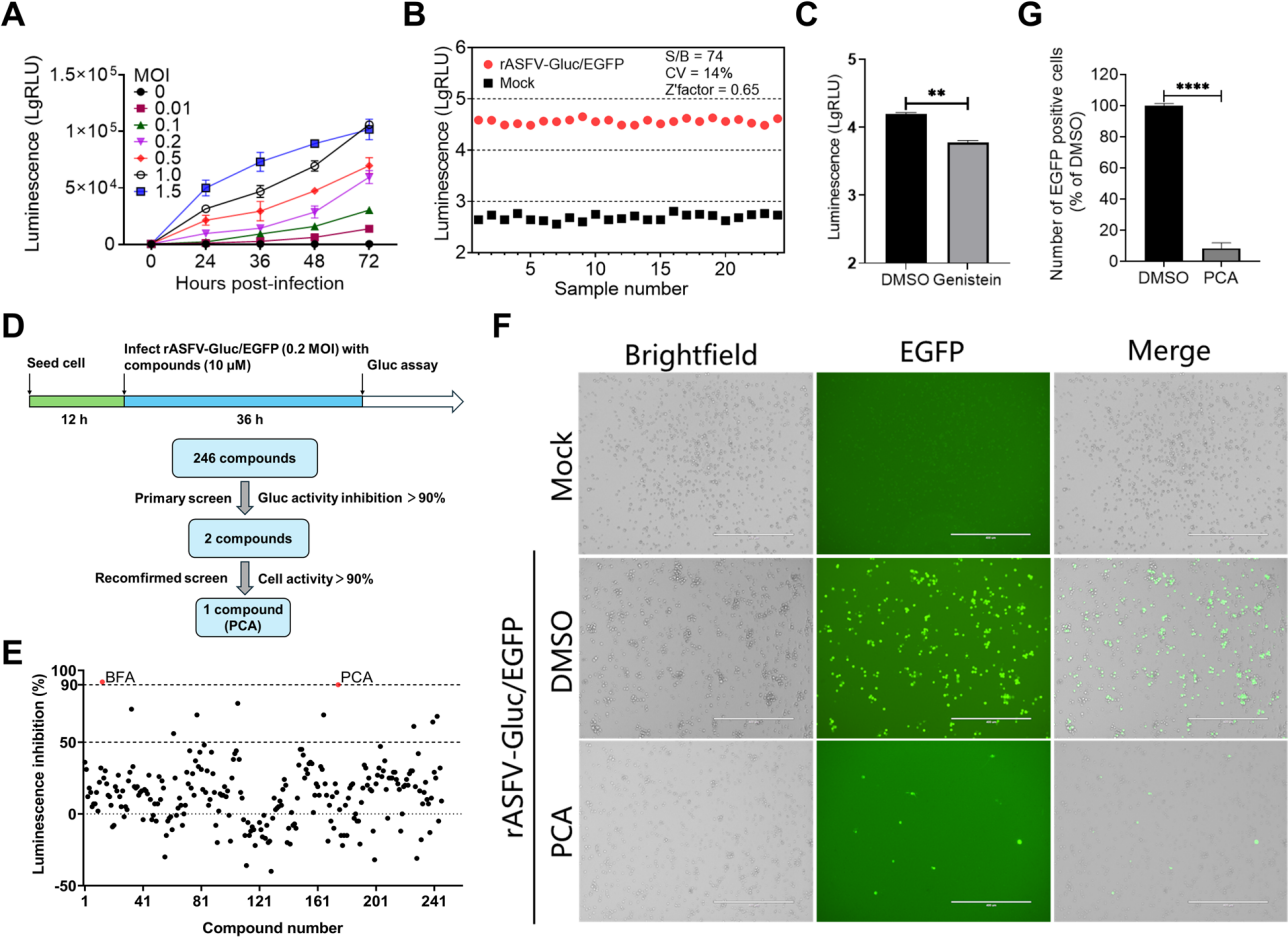
**Establishment of a high-throughput screen system using rASFV-Gluc/EGFP. A** Gluc activities in rASFV-Gluc/EGFP-infected PAMs. PAMs were infected with rASFV-Gluc/EGFP at a multiplicity of infection (MOI) of 0.01, 0.1, 0.2, 0.5, 1, or 1.5. Gluc activities were measured at 24, 36, 48, and 72 hours post-infection (hpi), and those of the mock-infected cells were regarded as background. The data are shown as the average ± standard deviation (*n* = 3). **B** High-throughput screening (HTS) assessment. PAMs were infected with rASFV-Gluc/EGFP at an MOI of 0.2 (*n* = 25). At 36 hpi, Gluc activities were measured, and the signal-to-background (S/B) ratio, coefficient of variation (CV), and Z-factor were calculated. The PAMs were measured for Gluc activities as background. **C** Antiviral compound screening assessment. PAMs were infected with rASFV-Gluc/EGFP (MOI = 0.2) in the presence of 10 μM genistein, and Gluc activities were measured at 36 hpi. The anti-ASFV activities of genistein were determined by comparing the Gluc activities of the genistein-treated cells to those of the DMSO-treated cells. **D** Schematic of the HTS workflow using the rASFV-Gluc/EGFP dual-reporter to identify potential anti-ASFV compounds. **E** A scatter plot representing the primary screening data of 246 small molecule compounds is displayed, with red dots indicating compounds with > 90% inhibition of ASFV. **F** and **G** Inhibitory effects of PCA on ASFV demonstrated by EGFP expression. PAMs were infected with rASFV-Gluc/EGFP (MOI = 0.2) in the presence of PCA or DMSO, and EGFP expression was observed by fluorescence microscopy at 36 hpi, with representative fluorescence images captured (**F**), and the number of EGFP-positive cells per field of view was measured using the ImageJ software (**G**). Scale bars = 400 μm. **P < 0.01; ****P < 0.0001.

To demonstrate the feasibility of using rASFV-Gluc/EGFP for HTS, we first optimized the HTS conditions by assessing Gluc activities in the PAMs infected with rASFV-Gluc/EGFP. The results revealed that Gluc activities were induced in the PAMs infected with rASFV-Gluc/EGFP at an MOI of 0.2 or higher at 24 hpi, and the number of RLUs significantly increased thereafter. When infected with rASFV-Gluc/EGFP at an MOI of 0.1 or 0.01, the cells failed to generate detectable Gluc signals at 24 hpi (Figure 4A). The MOI of rASFV-Gluc/EGFP was optimized as 0.2, and the measurement was performed at 36 hpi. As a result, the Z-factor, which indicates assay quality, was determined to be 0.65 (*n* = 25), indicating excellent suitability for HTS [24]. Additionally, the CV, reflecting signal deviation within the assay, was found to be 14%, which is well within the acceptable range (Figure 4B). The S/B ratio of 74 further confirms the suitability of the assay for HTS, ensuring robust signal detection relative to background noise. In testing the sensitivity of the dual-reporter virus to genistein, a known ASFV inhibitor [13], the results aligned with previous findings, demonstrating the inhibitory effects of genistein on ASFV replication (Figure 4C). Overall, these findings validate the feasibility of the HTS method based on rASFV-Gluc/EGFP for screening anti-ASFV compounds.

### HTS for anti-ASFV compounds

After the assay was optimized for screening compounds, we applied this method to screen a library consisting of 246 small molecule compounds (Figure 4D). In the primary screening, we evaluated the potential antiviral activities of these compounds against rASFV-Gluc/EGFP in PAMs. The detection conditions used for HTS were as follows: PAMs were infected with rASFV-Gluc/EGFP (MOI = 0.2) in the presence of the test compounds (10 μM) for 36 h. The compounds that exhibited a greater than 90% reduction in Gluc activities during the initial screening were selected for further confirmation. PCA and brefeldin A (BFA) exhibited promising antiviral activities in the primary screening (Figure 4E). Under fluorescence microscopy, we also observed that the expression of EGFP in rASFV-Gluc/EGFP was significantly suppressed by PCA (Figures 4F and G). These results indicate that PCA efficiently inhibits ASFV replication in PAMs.

### PCA inhibits ASFV replication

**Figure 5:**
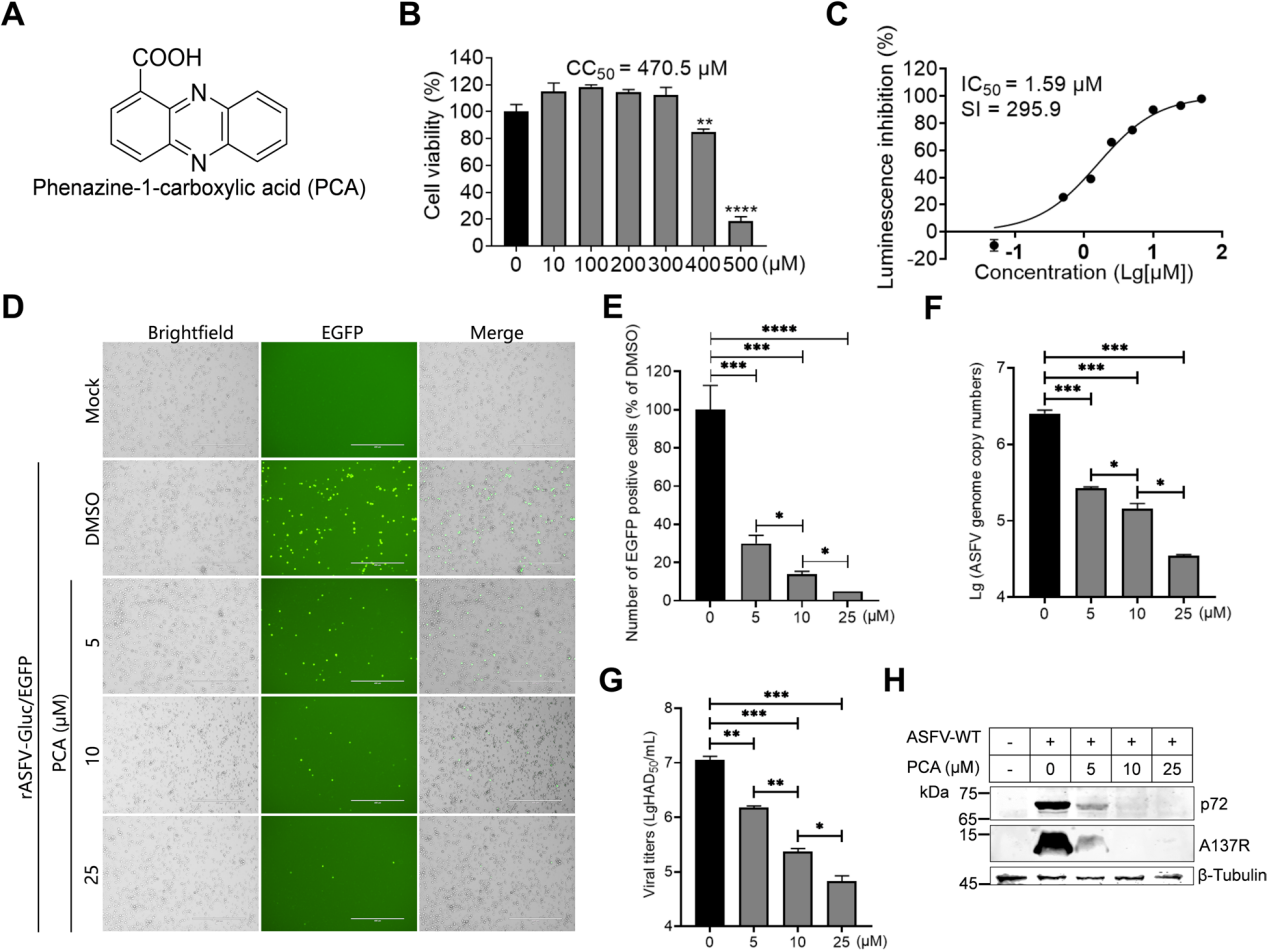
**Antiviral activities of phenazine-1-carboxylic acid (PCA) against ASFV. A** The chemical structure of PCA. **B** Half-cytotoxic concentration (CC_50_) of PCA in PAMs. The PAMs were exposed to varying concentrations of PCA, and cell viability was assessed using the CellTiter-Glo kit at 48 hours post-treatment. The CC_50_ was calculated using dose–response curves on GraphPad Prism. **C** The half-maximal inhibitory concentration (IC_50_) of PCA on ASFV. PAMs were infected with rASFV-Gluc/EGFP (MOI = 0.2) and treated with a specified concentration of PCA; luciferase activities were measured at 36 hours post-infection (hpi) to evaluate the IC_50_, which was calculated using nonlinear regression analysis of dose‒response curves on GraphPad Prism. **D** and **E** Anti-ASFV activities of PCA by EGFP expression assay. PAMs were infected with rASFV-Gluc/EGFP (MOI = 0.2) while being treated with PCA (0, 5, 10, or 25 μM), and EGFP expression was observed at 36 hpi, with representative fluorescence images captured (**D**), and the number of EGFP-positive cells per field of view was measured using the ImageJ software (**E**). Scale bars = 400 μm. **F** Inhibition of rASFV-Gluc/EGFP replication by PCA. ASFV genomic DNA was extracted from the PAMs infected with rASFV-Gluc/EGFP and then treated with a specified concentration of PCA (0, 5, 10, or 25 μM), after which the ASFV genome copies was measured by qPCR. **G** The antiviral effects of PCA on rASFV-Gluc/EGFP. PAMs were infected with rASFV-Gluc/EGFP (MOI = 0.2) and treated with a specified concentration of PCA (0, 5, 10, or 25 μM). The viral titres were determined by a hemadsorption (HAD) assay at 48 hpi. **H** Western blotting analysis of PCA-mediated inhibition of ASFV-WT. The protein expression levels of the p72 and A137R in the ASFV-WT-infected PAMs treated with different concentrations of PCA (0, 5, 10, or 25 μM) were analysed by western blotting. *P < 0.05; **P < 0.01; ***P < 0.001; ****P < 0.0001.

To ensure safe concentrations of PCA and BFA for PAMs, a secondary screening excluded the compounds that reduced cell viability by less than 90% at 10 μM. The results revealed that the CC_50_ of PCA on PAMs was 470.5 μM (Figures 5A and B). In comparison, the viability of the PAMs treated with 10 μM BFA was less than 10%, indicating that the inhibitory effects of BFA on ASFV are due to cytotoxicity (Additional file 6). Ultimately, only one compound, PCA, met the criteria. To study the antiviral activities of PCA against ASFV, we evaluated the IC_50_ of PCA against ASFV by quantifying the Gluc activities in the PAMs infected with rASFV-Gluc/EGFP. The results revealed that the IC_50_ of PCA against ASFV was 1.59 μM, which was significantly lower than the CC_50_ (Figures 5B and C). The SI was 295.9, indicating that PCA is a potential anti-ASFV compound (Figure 5C).

Next, the anti-ASFV effects of PCA were examined in PAMs by EGFP, qPCR, and viral titration assays. The results showed that PCA inhibited the expression of EGFP in a dose-dependent manner (Figures 5D and E). The results of the qPCR and viral titration assays also confirmed that PCA inhibited ASFV replication in a dose-dependent manner (Figures 5F and G). At a concentration of 25 μM, PCA can reduce the viral titre by more than 100 times. To confirm the antiviral activities of PCA against ASFV-WT, the effects of different doses of PCA (0, 5, 10, and 25 μM) on ASFV p72 and A137R protein expression were compared. The results revealed that the protein expression levels of ASFV p72 and A137R in the PAMs infected with ASFV-WT decreased with increasing PCA (Figure 5H). Overall, these results indicate that PCA effectively inhibits ASFV replication in PAMs in a dose-dependent manner, supporting its potential as a therapeutic agent against ASFV.

### The inhibitory effects of PCA on ASFV do not directly target the virus but rather influence the entire viral replication cycle

**Figure 6:**
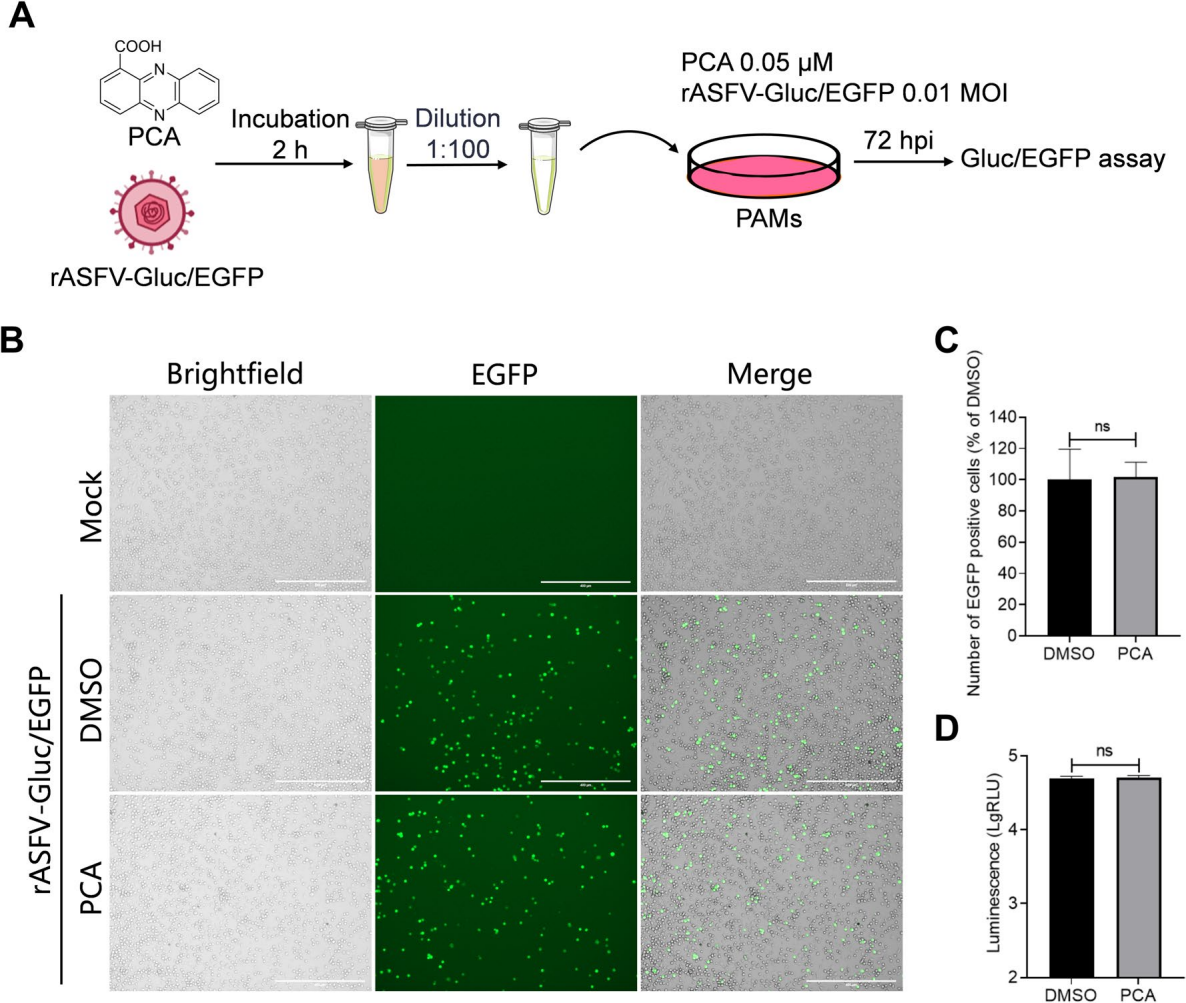
**PCA does not inactivate ASFV directly. A** Schematic diagram of the ASFV inactivation assay. **B** to **D** The inactivation effects of PCA on ASFV. PCA (10 μM) and rASFV-Gluc/EGFP (MOI = 1) were incubated at 37 °C, and after 2 h, the virus-PCA mixture was diluted 100-fold to infect PAMs. EGFP expression was observed at 36 hpi, with representative fluorescence images captured (**B**). The average number of EGFP-positive cells per field of view was measured using the ImageJ software (**C**). Scale bars = 400 μm. At 72 hpi, the supernatants were collected to assess Gluc activities (**D**). ns, not significant.

**Figure 7:**
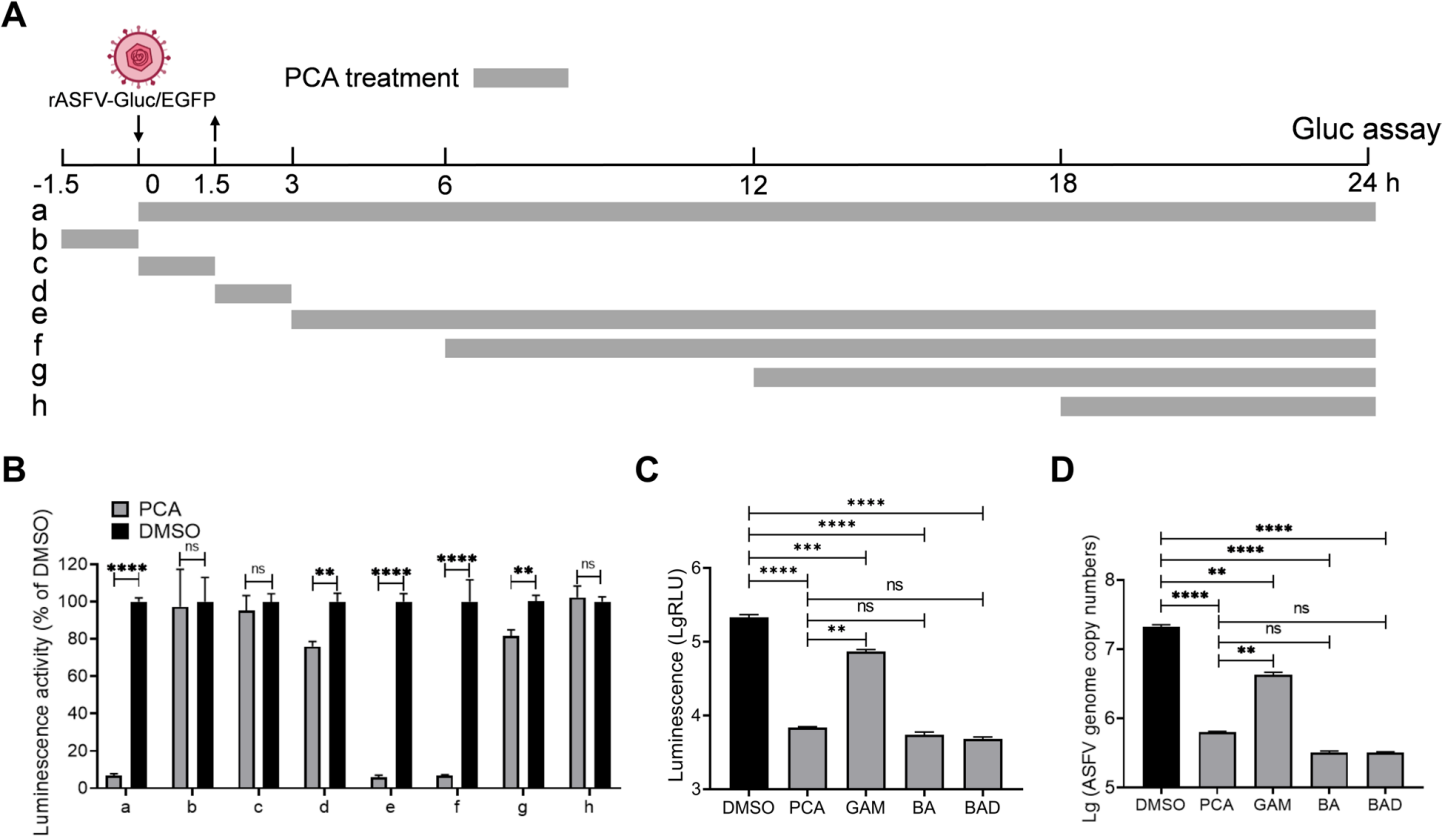
**PCA inhibits ASFV replication by targeting various stages of the ASFV life cycle. A** Schematic illustration of the time-of-addition experiment. **B** Effects of eight PCA treatments on the Gluc activities of rASFV-Gluc/EGFP. At 24 hpi, the supernatants were collected to detect Gluc activities, and the inhibitory effects of PCA were analysed, with DMSO serving as the negative control. **C** and **D** Comparison of the anti-ASFV inhibitory effects of PCA to those of other anti-ASFV molecules. PAMs were infected with rASFV-Gluc/EGFP (MOI = 0.2) and treated with 10 μM PCA, berbamine dihydrochloride (BAD), berbamine (BA), or gamithromycin (GAM). At 36 hpi, the supernatants from the PAMs were collected to detect Gluc activities (**C**), and the ASFV genome copies were detected by qPCR (**D**) to compare the inhibitory effects of PCA with those of other compounds. **P < 0.01; ***P < 0.001; ****P < 0.0001; ns, not significant.

To investigate whether PCA exerts its inhibitory effects through cells or the virus, we evaluated the effects of PCA on the inactivation of rASFV-Gluc/EGFP. In the virus inactivation assay, after co-incubating rASFV-Gluc/EGFP (MOI = 1) and PCA (10 μM) for 2 h, the ASFV-PCA mixture was diluted 1:100 for infection of PAMs, resulting in a final PCA concentration of 0.05 μM, at which time it did not inhibit ASFV replication (Figure 6A). Through the observation of EGFP fluorescence and the measurement of Gluc activities, we demonstrated that PCA did not directly inhibit the replication of ASFV (Figures 6B–D). To determine at which stage of the ASFV replication cycle PCA exerts its effects, we assessed the effect of PCA on ASFV at different time points (Figure 7A). PCA did not significantly inhibit ASFV replication when it was administered before infection, co-infection, or post-infection for 1.5 h of treatment. A longer incubation of PCA with ASFV resulted in more pronounced inhibition of ASFV replication (Figure 7B). These results indicate that PCA inhibits ASFV replication throughout the entire life cycle.

Finally, we compared the inhibitory effects of PCA with those of BAD, BA, and GAM at a concentration of 10 μM in PAMs, which have been reported to inhibit ASFV replication [25]. The results indicated that the inhibitory effects of PCA on ASFV were comparable to those of BA or BAD and greater than those of GAM (Figure 7C and D). Importantly, the CC_50_ values of BA, BAD, and GAM were less than 150 μM, whereas the CC_50_ values of PCA was greater than 450 μM, indicating that more options in terms of dosage exist. This would also be an advantage of PCA as a candidate anti-ASFV molecule.

## Discussion

ASFV is considered one of the major threats facing the global pig industry. No commercial ASF vaccines have been licenced, except in Vietnam. Therefore, alternative methods to prevent ASF are urgently needed. Some antiviral drugs have shown significant inhibitory effects in vitro. Discovering new antiviral compounds against ASFV, deciphering their mechanisms of action, and identifying new drug targets are highly important for the development of anti-ASFV drugs [4]. Establishing a novel and efficient virological assay is highly important for the study of ASFV biology and the development of anti-ASFV drugs.

In this study, we constructed a dual-reporter ASFV that co-expresses the *Gluc* and *EGFP* reporter genes. Importantly, rASFV-Gluc/EGFP and ASFV-WT exhibited no significant differences in terms of viral replication, virus particle morphology, or hemadsorption activity. This preservation of viral integrity highlights the feasibility and utility of the dual-reporter system for ASFV research. We investigated the relationship between ASFV genome copies and Gluc activities, revealing a robust linear correlation. This correlation not only validates the reliability of Gluc as a quantitative tool for assessing ASFV replication dynamics but also underscores its potential for HTS applications.

Furthermore, with the help of NGS, Gluc, and EGFP assays, we confirmed that the virus remained genetically stable after 20 consecutive passages. With rASFV-Gluc/EGFP, EGFP could be used to identify infected cells in vitro, whereas Gluc represents a better option to provide quantified levels of replication. Both reporter genes can be used as valid surrogates of ASFV infection since their levels of expression correlate with those of viral replication. By using two distinct reporter systems, the antiviral or neutralizing activities and inactivating effects of antivirals, antibodies, or disinfectants against ASFV can be effectively assessed. Importantly, the simultaneous use of two distinct reporter systems effectively reduces false positives resulting from the quenching of fluorescence or bioluminescence, thereby increasing the accuracy of the experimental results.

Replication-competent reporter viruses that produce bioluminescent or fluorescent signals have been constructed for a variety of viruses and proven immensely helpful for HTS, monitoring viral replication dynamics in vitro and in vivo, and studying basic or clinical virology [6, 26, 27]. Recently, Li et al. reported that they successfully constructed a dual-reporter virus (rASFV-Gluc-GFP) co-expressing *Gluc* and *GFP* on the basis of the deletion of the ASFV *K145R* gene [8]. They utilized the properties of Gluc and GFP for HTS and drug studies and demonstrated that triapine and cytarabine hydrochloride had significant anti-ASFV activity in vitro [8]. Although deletion of the *K145R* gene has not been found to affect ASFV replication, it may pose unknown risks in practical detection processes. In this study, we constructed rASFV-Gluc/EGFP without gene deletions, and the data demonstrated that the insertion of the reporter genes did not affect the virus. By retaining the complete viral genome, the dual-reporter virus not only provides a more comprehensive model for studying ASFV but also mitigates potential risks associated with genetic modifications. This method enhances the reliability of the virological assay and strengthens the foundation for future studies aimed at understanding ASFV pathogenesis. rASFV-Gluc/EGFP constitutes a versatile and simple tool for the rapid study of viral dynamics and the development of anti-ASFV compound screening platforms.

PCA, a natural metabolite produced by microorganisms such as *Pseudomonas* and *Streptomyces*, is known for its potent antifungal activities against various plant pathogens [28–31]. While its effects on fungi are well documented, its potential against viruses, including ASFV, remains relatively unexplored. In this study, on the basis of the excellent genetic stability of rASFV-Gluc/EGFP and the high-throughput properties of Gluc, we successfully established and evaluated an HTS method for screening anti-ASFV compounds. This HTS method was evaluated using metrics such as the Z-factor, and its effectiveness was verified using genistein. Using this approach, we screened a library of 246 small molecule compounds and identified two candidates with inhibition rates exceeding 90%. Subsequent cytotoxicity assays revealed that only one compound, PCA, inhibited ASFV by over 90% at a concentration of 10 μM, which was within a safe concentration range. Furthermore, we demonstrated that PCA dose-dependently suppressed ASFV replication in vitro. Nevertheless, PCA does not directly inactivate ASFV, and its anti-ASFV activity is achieved by inhibiting ASFV replication throughout the entire viral life cycle.

Although the evaluation of anti-ASFV compounds in this study was conducted in 96-well cell culture plates, it can be performed in a 384-well format for convenient and efficient HTS. Importantly, the dual-reporter virus rASFV-Gluc/EGFP enables the flexible use of one or both reporter genes to further validate anti-ASFV activity. Nevertheless, it should be noted that PCA does not directly inactivate ASFV. Rather, its anti-ASFV ability is achieved through a combination of effects on all stages of viral replication.

The mechanism by which PCA inhibits ASFV is currently unclear and warrants further investigation. It has been demonstrated that PCA can function as a redox agent, disrupting the redox balance of *Xanthomonas oryzae* pv. *oryzae* (*Xoo*), which leads to the reduced activities of catalase and superoxide dismutase, resulting in the accumulation of reactive oxygen species (ROS) and altered carbohydrate metabolism, thereby reducing the energy production and nutrient absorption capacity of *Xoo* [32]. Furthermore, PCA can penetrate hyphal cells of *Botrytis cinerea*, resulting in the accumulation of ROS and a significant reduction in the hyphal network along with morphological changes [33]. The PCA-producing *P. fluorescens* strain LBUM223 has been shown to repress the expression of virulence-associated genes (*txtA* and *txtC*) in the bacterial potato pathogen *Streptomyces scabies*, thereby reducing common potato scab disease symptoms [34–36]. Furthermore, PCA can serve as an interspecies regulator of antibiotic resistance, dramatically changing the susceptibility of diverse bacteria to clinical antibiotics [37]. These mechanisms suggest that PCA may exert inhibitory effects on ASFV through similar pathways.

Despite the current lack of research on the effects of PCA on viruses, relevant data indicate that the oral half-maximal lethal dose (LD_50_) of PCA is 369 mg/kg for male rats and 271 mg/kg for female rats. These findings suggest that PCA has low toxicity to rats [38]. Consequently, PCA may also exhibit low toxicity when orally administered to pigs or other animals. Therefore, PCA could be considered for the development of biological agents, incorporation into feed, or combination with other antiviral drugs to enhance efficacy, improve therapeutic outcomes, and minimize side effects. Further investigation into the inhibitory mechanisms of PCA against ASFV is crucial for identifying new targets for the prevention and control of ASF. Insights gained from these studies may provide a basis for the application of PCA and contribute to the development of broad-spectrum antivirals against other viruses.

In summary, the biological characteristics of rASFV-Gluc/EGFP are not significantly different from those of ASFV-WT. Utilizing rASFV-Gluc/EGFP, we established an HTS method and screened 246 small molecule compounds, leading to the identification of PCA as a potent anti-ASFV molecule. Therefore, PCA is a promising candidate for the development of anti-ASFV drugs.

The dual-reporter virus rASFV-Gluc/EGFP is a dependable, precise, simple, and efficient tool for virological assays. Using rASFV-Gluc/EGFP, we conducted HTS of 246 small-molecule compounds and identified PCA as a significant inhibitor of ASFV replication in PAMs. This discovery opens new avenues for the development of ASF prevention strategies.

## Supplementary Information

**Additional file 1.** **The compound library contains 246 small-molecule compounds.**

**Additional file 2**. **The primers used in this study.**

**Additional file 3.** **Generation of the transfer vector pOK12-Gluc/EGFP and the dual-reporter virus rASFV-Gluc/EGFP**. (A) Generation of the transfer vector pOK12-Gluc/EGFP. The genomic fragment covering the left homology arm, p72-Gluc, p72-EGFP, and the right homology arm were amplified by PCR and analysed by agarose gel electrophoresis. (B) Gluc assay. HEK293T cells were transfected with the transfer vector pOK12-Gluc/EGFP and then infected with ASFV-P121 (MOI = 5), and the Gluc activities in the supernatants were measured at 24 hours post-infection (hpi). (C) Observation of EGFP expression. The pOK12-Gluc/EGFP transfer vector was transfected into HEK293T cells, which were inoculated with ASFV-P121 (MOI = 5), and the EGFP expression was observed by fluorescence microscopy at 24 hpi. (D) Schematic diagram of rASFV-Gluc/EGFP generation and purification. **P < 0.01.

**Additional file 4.** **Next-generation sequencing analysis of rASFV-Gluc/EGFP**.

**Additional file 5.** **The genetic stability of rASFV-Gluc/EGFP**. (A) Schematic diagram of continuous passaging of rASFV-Gluc/EGFP. (B to D) Hemadsorption and fluorescence assays of P10. rASFV-Gluc/EGFP was passaged in PAMs for 20 consecutive passages, with EGFP expression and hemadsorption observed at P10 by fluorescence microscopy (B). The number of EGFP-positive cells (C) or hemadsorption-positive cells (D) per field of view was measured using the ImageJ software. Red arrows indicate hemadsorption. Scale bars = 400 μm. ns, not significant.

**Additional file 6.** **The cytotoxicity of brefeldin A (BFA) to PAMs and its inhibitory effects on ASFV. **(A) The chemical structure of BFA. (B) The cytotoxicity of BFA to PAMs. (C) Inhibitory effects of BFA on rASFV-Gluc/EGFP. PAMs were infected with rASFV-Gluc/EGFP (MOI = 0.2) and treated with BFA (0, 0.01, or 0.05 μM), and Gluc activities were assayed at 36 hpi. *P < 0.1; ****P < 0.0001; ns, not significant.

## Data Availability

The datasets used and/or analysed during the current study are available from the corresponding author upon reasonable request. The dataset supporting the conclusions of this article is included within the article (and its supplementary information files).
