# Supplementary material for: A novel high-throughput screen identifies phenazine-1-carboxylic acid as an inhibitor of African swine fever virus replication in primary porcine alveolar macrophages
Source: Vet Res. 2025 Feb 8;56:37. doi: 10.1186/s13567-025-01467-2 (PMC11806816; doi:10.1186/s13567-025-01467-2)
Supplement: Supplementary file 1 — Additional file 1. The compound library contains 246 small-molecule compounds. [file 13567_2025_1467_MOESM1_ESM.docx]

**Additional file 1 The compound library containing 246 small molecule compounds.**

| Number | Name | Formula | Molecular weight (Da) | Type |
| --- | --- | --- | --- | --- |
| 1 | 4-Methylbenzophenone | C_14_H_12_O | 196.25 | Polyketones |
| 2 | 2,5-Anhydro-D-mannitol | C_6_H_12_O_5_ | 164.16 | Carbohydrates |
| 3 | (2R,3R)-4-Methyl-1-phenyl-  2,3-pentanediol | C_12_H_18_O_2_ | 194.27 | Polyketones |
| 4 | α-(1-Hydroxycyclohexyl)  benzenemethanol | C_13_H_18_O_2_ | 206.29 | Polyketones |
| 5 | 4-Methyl-1-phenyl-2,3-hexanediol | C_13_H_20_O_2_ | 208.30 | Polyketones |
| 6 | (1R)-1-(3,5-Dimethoxyphenyl)-1,2-ethanediol | C_10_H_14_O_4_ | 198.22 | Polyketones |
| 7 | 3-(Phenylmethyl)-2(3H)-benzofuranone | C_15_H_12_O_2_ | 224.26 | Polyketones |
| 8 | Benzyl α-L-rhamnopyranoside | C_13_H_18_O_5_ | 254.28 | Polyketones |
| 9 | 1-Phenylcyclohexanol | C_12_H_16_O | 176.26 | Polyketones |
| 10 | (2R)-1-Cyclopentyl-2-hydroxy-2-phenylethanone | C_13_H_16_O_2_ | 204.27 | Polyketones |
| 11 | (+)-2-[Hydroxy(2-methyl-1-oxopropyl)amino]-3-methylbutanamide | C_9_H_18_N_2_O_3_ | 202.25 | Polyketones |
| 12 | (3β,5α,6α,22E)-Ergosta-7,22-diene-3,5,6-triol | C_28_H_46_O_3_ | 430.67 | Terpenes |
| 13 | Brefeldin A | C_16_H_24_O_4_ | 280.36 | Macrolides |
| 14 | Azelaic acid | C_9_H_16_O_4_ | 188.10 | Fatty acids |
| 15 | (4S)-(2E)-4-hydroxy-  2-nonenoic acid | C_9_H_16_O_3_ | 171.10 | Fatty acids |
| 16 | Cerevisterol | C_28_H_46_O_3_ | 430.66 | Sterols |
| 17 | 1-Hydroxycyclohexyl phenyl ketone | C_13_H_16_O_2_ | 204.27 | Polyketides |
| 18 | 2,5-Dihydro-4-hydroxy-5-oxo-3-phenyl-2-furanpropanoic acid | C_13_H_12_O_5_ | 248.23 | Polyketides |
| 19 | Ethyl 2-acetyl-  3,5-dihydroxybenzeneacetate | C_12_H_14_O_5_ | 238.24 | Phenylacetic acids |
| 20 | 15-Deacetylated citreohybridone E | C_28_H_38_O_8_ | 502.26 | Meroterpenoids |
| 21 | 9-Hydroxyhelminthosporol | C_15_H_24_O_2_ | 236.18 | Sesquiterpenes |
| 22 | 1,6,11-Eudesmanetriol; (1a,6b)-form | C_15_H_28_O_3_ | 256.20 | Sesquiterpenes |
| 23 | (4S)-2,6,10-Bisaboratrien-4-ol-1-one | C_15_H_22_O_2_ | 234.16 | Sesquiterpenes |
| 24 | Penioxalicin | C_19_H_26_O_6_ | 350.17 | Diterpenes |
| 25 | Talarobicin A | C_21_H_34_O_4_ | 350.25 | Diterpenes |
| 26 | Penitholabene | C_19_H_26_O_5_ | 334.18 | Diterpenes |
| 27 | Eupenicisirenins B | C_10_H_12_O_4_ | 196.07 | Norsesquiterpenoids |
| 28 | Peniterpenoid A | C_15_H_20_O_5_ | 280.13 | Diterpenes |
| 29 | Eupenicisirenins A | C_15_H_22_O_3_ | 250.16 | Diterpenes |
| 30 | Eupenicisirenins D | C_12_H_16_O_4_ | 224.10 | Norsesquiterpenoids |
| 31 | Eupenicisirenins O | C_15_H_20_O_4_ | 264.13 | Diterpenes |
| 32 | Cyclo-(R-Isoleu-R-Leu) | C_12_H_22_N_2_O_2_ | 226.17 | Diketopiperazines |
| 33 | Cyclo-(L-Phe-N-ethyl-L-Glu) | C_16_H_20_N_2_O_4_ | 304.14 | Diketopiperazines |
| 34 | Cyclo-(L-trans-Hyp-L-Phe) | C_14_H_16_N_2_O_2_ | 244.12 | Diketopiperazines |
| 35 | Callyspongidipeptide A | C_11_H_18_N_2_O_3_ | 226.13 | Diketopiperazines |
| 36 | Brasilamides E | C_15_H_19_NO_2_ | 245.14 | Sesquiterpenoid |
| 37 | Theissenisochromanone | C_10_H_9_O_5_ | 209.04 | Polyketides |
| 38 | Tazettone A | C_16_H_16_O_4_ | 272.10 | Flavones |
| 39 | Liquiritigenin | C_15_H_12_O_4_ | 256.07 | Flavanones |
| 40 | 6-Methylaromadendrin | C_16_H_14_O_6_ | 302.08 | Flavanones |
| 41 | Poriol | C_16_H_14_O_5_ | 286.08 | Flavanones |
| 42 | Naringenin | C_15_H_12_O_5_ | 272.07 | Flavanones |
| 43 | 8-Methylnaringenin | C_16_H_14_O_4_ | 270.28 | Flavanones |
| 44 | 3′,7-Dihydroxy-4′-methoxyflavan | C_16_H_16_O_4_ | 272.10 | Flavans |
| 45 | 7,4’-Dihydroxy-8-methylflavan | C_16_H_16_O_3_ | 256.11 | Flavans |
| 46 | 4′,7-Dihydroxyflavan | C_15_H_14_O_3_ | 242.27 | Flavans |
| 47 | 7-Hydroxy-2-methylchromone | C_10_H_8_O_3_ | 176.17 | Chromones |
| 48 | 5-Didroxy-7,8-dimethoxy-2-methylchromone | C_12_H_12_O_5_ | 236.22 | Chromones |
| 49 | 8-Methoxy-5,7-dihydroxy-2-methylchromone | C_11_H_10_O_5_ | 222.19 | Chromones |
| 50 | Noreugenin | C_10_H_8_O_4_ | 192.17 | Chromones |
| 51 | 3-Epimacronine | C_18_H_19_NO_5_ | 329.13 | Homolycorines |
| 52 | Secoisolariciresinol | C_20_H_26_O_6_ | 362.17 | Flavones |
| 53 | 7-(3-Ethoxy-5-methoxyphenyl)propane-7,8,9-triol | C_12_H_18_O_5_ | 242.12 | Flavones |
| 54 | 2′,6′-Dimethoxy-  4′-hydroxyacetophenone | C_1_OH_12_O_4_ | 196.07 | Ketones |
| 55 | 5-Hydroxymethylfuran-3-carboxylic acid | C_6_H_6_O_4_ | 142.02 | Carboxylic acids |
| 56 | 4′-(4,5-Dimethyl-1,3-dioxolan-2-yl)  methylphenol | C_12_H_16_O_3_ | 208.11 | Phenols |
| 57 | 4-Hydroxybenzaldehyde | C_7_H_6_O_2_ | 122.12 | Phenols |
| 58 | 2′,4′,6′-Trimethoxyacetophenone | C_11_H_14_O_4_ | 210.23 | Ketones |
| 59 | Hypercohone F | C_38_H_50_O_5_ | 586.37 | PPAPs |
| 60 | Hypercohin E | C_35_H_52_O_5_ | 552.38 | PPAPs |
| 61 | Hyperacmosin J | C_30_H_44_O_5_ | 484.32 | PPAPs |
| 62 | Hyphenrone T | C_35_H_52_O_6_ | 568.38 | PPAPs |
| 63 | Hyphenrone U | C_35_H_52_O_6_ | 568.38 | PPAPs |
| 64 | Hyperscabin A | C_34_H_52_O_5_ | 540.38 | PPAPs |
| 65 | Furoadhyperforin | C_36_H_54_O_5_ | 566.40 | PPAPs |
| 66 | Hyphenrone W | C_35_H_52_O_6_ | 568.38 | PPAPs |
| 67 | Hypericumoxide C | C_35_H_54_O_7_ | 586.39 | PPAPs |
| 68 | Wilsonglucinol G | C_30_H_44_O_5_ | 484.32 | PPAPs |
| 69 | Betulinic acid | C_30_H_48_O_3_ | 456.36 | Triterpenes |
| 70 | Wilsonglucinol D | C_30_H_44_O_5_ | 484.32 | PPAPs |
| 71 | Hirsutofolin A | C_30_H_44_O_5_ | 484.32 | PPAPs |
| 72 | Wilsonglucinol E | C_31_H_46_O_5_ | 489.33 | PPAPs |
| 73 | Attenuatumione A | C_31_H_46_P_5_ | 489.33 | PPAPs |
| 74 | Hyperacmosin G | C_33_H_42_O_5_ | 518.30 | PPAPs |
| 75 | Hypersampsone O | C_33_H_40_O_5_ | 516.29 | PPAPs |
| 76 | Sampsonione Q | C_33_H_40_O_5_ | 516.29 | PPAPs |
| 77 | Hypericumoxide M | C_35_H_54_O_7_ | 586.39 | PPAPs |
| 78 | Hypersampsone Q | C_33_H_42_O_5_ | 518.30 | PPAPs |
| 79 | Hirsutusal A | C_30_H_44_O_5_ | 484.32 | PPAPs |
| 80 | Sampsonione B | C_35_H_46_O_6_ | 562.33 | PPAPs |
| 81 | 8-Benzoyl-4α-(1-hydroxy-  methylethyl)-7,7-dimethyl-1,3-di  (3-methyl-2-butenyl)tricyclo  [4.3.1.13,8]undecane-2,9,11-trione | C_33_H_42_O_5_ | 518.30 | PPAPs |
| 82 | Furohyperforin | C_35_H_52_O_5_ | 552.38 | PPAPs |
| 83 | Sampsonione K | C_38_H_50_O_5_ | 586.37 | PPAPs |
| 84 | Hypericumoxide I | C_35_H_57_O_7_ | 586.39 | PPAPs |
| 85 | Kiiacylphnols A | C_35_H_52_O_5_ | 568.38 | PPAPs |
| 86 | Oxepahyperforin | C_35_H_52_O_5_ | 552.38 | PPAPs |
| 87 | Hyperacmosin F | C_35_H_52_O_5_ | 552.38 | PPAPs |
| 88 | Hirsutusal C | C_31_H_46_O_5_ | 498.33 | PPAPs |
| 89 | Hyperhomanoon A | C_31_H_44_O_5_ | 496.32 | PPAPs |
| 90 | Hyperhomanoon B | C_30_H_42_O_5_ | 482.30 | PPAPs |
| 91 | Hyperhomanoon D | C_32_H_38_O_6_ | 518.27 | PPAPs |
| 92 | Hyperhomanoon E | C_32_H_38_O_6_ | 518.27 | PPAPs |
| 93 | Patumantane C | C_29_H_44_O_4_ | 456.32 | PPAPs |
| 94 | Patumantane D | C_31_H_46_O_6_ | 514.33 | PPAPs |
| 95 | Hyperadaman A | C_33_H_38_O_6_ | 530.27 | PPAPs |
| 96 | Hyperadaman B | C_33_H_40_O_6_ | 532.28 | PPAPs |
| 97 | Hyperadaman C | C_33_H_40_O_6_ | 532.28 | PPAPs |
| 98 | Hyperadaman D | C_33_H_40_O_6_ | 532.28 | PPAPs |
| 99 | Hyperadaman E | C_33_H_40_O_6_ | 532.28 | PPAPs |
| 100 | Hyperadaman F | C_33_H_42_O_6_ | 534.30 | PPAPs |
| 101 | Hyperadaman G | C_30_H_44_O_6_ | 500.31 | PPAPs |
| 102 | Cumilcinol A | C_30_H_44_O_7_ | 516.31 | PPAPs |
| 103 | Cumilcinol B | C_34_H_42_O_6_ | 546.30 | PPAPs |
| 104 | Cumilcinol C | C_31_H_46_O_6_ | 514.33 | PPAPs |
| 105 | Cumilcinol F | C_31_H_46_O_5_ | 498.33 | PPAPs |
| 106 | Cumilcinol E | C_27_H_38_O_5_ | 442.27 | PPAPs |
| 107 | Cumilcinol I | C_29_H_44_O_4_ | 456.32 | PPAPs |
| 108 | Cumilcinol G | C_33_H_42_O_6_ | 534.30 | PPAPs |
| 109 | Cumilcinol H | C_30_H_42_O_5_ | 482.30 | PPAPs |
| 110 | Dioxasampsone A | C_33_H_42_O_6_ | 534.30 | PPAPs |
| 111 | Dioxasampsone B | C_33_H_42_O_7_ | 550.29 | PPAPs |
| 112 | Soniiglucinol C | C_29_H_44_O_4_ | 456.32 | PPAPs |
| 113 | Ent-4(15)-eudesmene-1β,6α-diol | C_15_H_26_O_2_ | 238.19 | Sesquiterpenes |
| 114 | Oleanoic acid | C_30_H_48_O_3_ | 456.36 | Triterpenes |
| 115 | 2,3-Butanediol | C_4_H_10_O_2_ | 90.12 | Fatty acids |
| 116 | 3-Methoxy-5-methylphenol | C_8_H_10_O_2_ | 138.16 | Polyketones |
| 117 | Notoamide C | C_26_H_31_N_3_O_4_ | 449.23 | Prenylated  indole alkaloids |
| 118 | 17-O-Ethylnotoamide M | C_28_H_35_N_3_O_5_ | 493.60 | Prenylated  indole alkaloids |
| 119 | Notoamide Q | C_27_H_33_N_3_O_5_ | 479.60 | Prenylated  indole alkaloids |
| 120 | 17-Epi-notoamide M | C_26_H_31_N_3_O_5_ | 465.23 | Prenylated  indole alkaloids |
| 121 | Norgeamides D | C_26_H_31_N_3_O_5_ | 465.23 | Prenylated  indole alkaloids |
| 122 | N19-Methyl-notoamide | C_26_H_31_N_3_O_4_ | 449.23 | Prenylated  indole alkaloids |
| 123 | Asperochramide A | C_26_H_33_N_3_O_4_ | 451.25 | Prenylated  indole alkaloids |
| 124 | Versicolamide C | C_26_H_29_N_3_O_5_ | 463.21 | Prenylated  indole alkaloids |
| 125 | Kipukasins G | C_19_H_22_N_2_O_9_ | 422.13 | Nucleosides |
| 126 | Kipukasins E | C_19_H_22_N_2_O_9_ | 422.13 | Nucleosides |
| 127 | (3S,12aS)-2,3,6,7,12,12a-hexahydro-3-(1-Methylethyl)  pyrazino[1′,2′:1,6]  pyrido[3,4-b]indole-1,4-dione | C_18_H_21_N_3_O_2_ | 311.16 | Diketopiperazine-type alkaloids |
| 128 | (3R,6S)-3-(1-Methylethyl)-6-(2-methylpropyl)-2,5-piperazinedione | C_11_H_20_N_2_O_2_ | 212.29 | Diketopiperazines |
| 129 | 2,5-Piperazinedione,-benzyl-6-isobutyl-, cis- | C_15_H_20_N_2_O_2_ | 260.33 | Diketopiperazines |
| 130 | Perlolyrine | C_16_H_12_N_2_O_2_ | 264.28 | Indole alkaloids |
| 131 | Glulisine A | C_11_H_17_N_3_O_2_ | 223.27 | Amino acid  derivatives |
| 132 | 5-(5H-Cyclopenta[c]cinnolin-3-yl)-2-furanmethanol | C_16_H_12_N_2_O_2_ | 264.28 | Indole alkaloids |
| 133 | Acetamide | C_10_H_13_NO | 163.09 | Alkaloids |
| 134 | Seco-((S)-Pro-(R)-Val) | C_10_H_19_N_3_O_2_ | 213.28 | Diketopiperazines |
| 135 | Stoloniferol B | C_12_H_14_O_2_ | 222.09 | Polyketones |
| 136 | 3,4,5,7-Tetramethylisochromane-3,6,8-triol | C_13_H_18_O_4_ | 238.12 | Polyketones |
| 137 | (3R,4S)-3,4-Dihydro-6,8-dihydroxy-3,4,5,7-tetramethyl-1H-2-benzopyran-1-one | C_13_H_16_O_4_ | 236.10 | Polyketones |
| 138 | Coniochaetone B | C_13_H_12_O_4_ | 232.07 | Polyketones |
| 139 | Xanthone | C_16_H_16_O_7_ | 320.09 | Polyketones |
| 140 | Coniochaetone E | C_14_H_14_O_4_ | 246.09 | Polyketones |
| 141 | Rhodocoranes L | C_14_H_20_O_4_ | 252.14 | Polyketones |
| 142 | Citrinal A | C_15_H_22_O_5_ | 282.15 | Polyketones |
| 143 | Penicillide | C_20_H_22_O_6_ | 358.14 | Polyketones |
| 144 | Penicitrinone F | C_24_H_26_O_5_ | 394.18 | Polyketones |
| 145 | Spirocitrinols A | C_26_H_32_O_6_ | 440.22 | Polyketones |
| 146 | Isocyclocitrinol A | C_25_H_36_O_4_ | 400.26 | Steroids |
| 147 | Norcyclocitrinoic acid A | C_23_H_30_O_4_ | 370.21 | Steroids |
| 148 | 23-Oxoneocyclocitrinol | C_25_H_34_O_4_ | 398.25 | Steroids |
| 149 | Neocyclocitrinol B | C_23_H_32_O_3_ | 356.24 | Steroids |
| 150 | Cyclocitrinol | C_21_H_28_O_3_ | 328.20 | Steroids |
| 151 | Ergosterol | C_28_H_44_O | 396.34 | Steroids |
| 152 | Sorbicatechol A | C_22_H_24_O_5_ | 368.16 | Polyketones |
| 153 | Trichodimerol | C_28_H_32_O_8_ | 496.21 | Polyketones |
| 154 | Dihydrotrichodimerol | C_28_H_34_O_8_ | 498.23 | Polyketones |
| 155 | Pyripyropene A | C_36_H_37_NO_10_ | 583.24 | Alkaloids |
| 156 | L-Pyroglutamy-L-phenylalanine | C_15_H_18_N_2_O_4_ | 290.13 | Alkaloids |
| 157 | N-(2-Phenylethyl)acetamide | C_10_H_13_NO | 163.10 | Alkaloids |
| 158 | Callyspongidipeptide A | C_11_H_18_N_2_O_3_ | 226.13 | Diketopiperazines |
| 159 | Cyclo-(L-Leu-L-Pro) | C_11_H_18_N_2_O_2_ | 210.14 | Diketopiperazines |
| 160 | Cyclo-(Leucylvalyl) | C_11_H_20_N_2_O_2_ | 212.15 | Diketopiperazines |
| 161 | 3-Isobutyl-tetrahydro-imidazo[1,2- a ]pyridine-2,5-dione | C_11_H_18_N_2_O_2_ | 210.14 | Diketopiperazines |
| 162 | 3-Methoxy-1-methyl-4(1H)- quinolone | C_11_H_11_NO_2_ | 189.08 | Alkaloids |
| 163 | Tryprostatin B | C_21_H_25_N_3_O_2_ | 351.19 | Diketopiperazines |
| 164 | Purpuride D | C_22_H_31_NO_5_ | 389.22 | Alkaloids |
| 165 | α,β-Dehydromonacolin S | C_23_H_34_O_6_ | 406.24 | Polyketones |
| 166 | Aceto-vanillone | C_9_H_10_O_3_ | 166.06 | Polyketones |
| 167 | (6S,7ar)-5,6,7,7a-Tetrahydro-6-hydroxy-4,4,7a-trimethyl-2(4H)-benzofuranone | C_11_H_16_O_3_ | 196.11 | Polyketones |
| 168 | Benzeneacetic acid | C_12_H_16_O_4_ | 224.10 | Polyketones |
| 169 | Paeciloxazine | C_29_H_38_N_2_O_8_ | 542.26 | Sesquiterpenoid alkaloids |
| 170 | Sugikurojinols A | C_15_H_22_O_2_ | 234.16 | Sesquiterpenoids |
| 171 | 2-Cyclohexen-1-one, 4,5,6-trihydroxy-5-methyl-2-(5-methyl-1-methylene-4-hexen-1-yl)-, (4R,5S,6R)-rel-(+) | C_15_H_22_O_4_ | 266.15 | Sesquiterpenoids |
| 172 | Cyclohexanone, 6-(1,5-dimethyl-4-hexen-1-ylidene)-2,3,4-trihydroxy-3-methyl-, (2R,3S,4R,6E)-rel-(+)- | C_15_H_24_O_4_ | 268.17 | Sesquiterpenoids |
| 173 | Phomaligol A1 | C_14_H_22_O_6_ | 286.14 | Polyketones |
| 174 | Latifolicinin B | C_12_H_16_O_3_ | 208.11 | Polyketones |
| 175 | Phenazine-1-carboxylic acid | C_13_H_8_N_2_O_2_ | 224.21 | Alkaloids |
| 176 | Ethyl ferulate | C_12_H_14_O_4_ | 222.09 | Polyketones |
| 177 | 2-Methyl-, (8R)-7,8-dihydro-5-methoxy-6,8-dimethyl-7-oxo-8-quinolinyl ester | C_17_H_21_NO_4_ | 303.15 | Alkaloids |
| 178 | Butanoic acid | C_15_H_25_NO_5_ | 311.17 | Alkaloids |
| 179 | Brevianamide F | C_16_H_17_N_3_O_2_ | 283.13 | Diketopiperazines |
| 180 | 1-Methyl-4-quinolone | C_10_H_9_NO | 156.07 | Quinnolones |
| 181 | Hydroxyphenylacetic acid | C_9_H_10_O_4_ | 182.06 | Polyketones |
| 182 | Aspergerthin acid A | C_10_H_14_O_4_ | 198.09 | Monoterpenes |
| 183 | Jbir-113 | C_31_H_41_N_5_O_7_ | 595.30 | Macrolide lactams |
| 184 | Jbir-115 | C_30_H_35_N_5_O_7_ | 581.28 | Macrolide lactams |
| 185 | Rel-(-)-(5R,6R)-6-[(2Z,4Z,6E,11E,13Z,19E)-10,16-Dihydroxy-11,19-dimethyl-2,4,6,1 | C_18_H_28_O_3_ | 480.32 | Fatty acids |
| 186 | (10E,12E,14S)-14-Pentyloxacyclotetradeca-10,12-diene-2,9-dione | C_18_H_28_O_3_ | 292.20 | Macrolides |
| 187 | Methyl linoleate | C_19_H_34_O_2_ | 294.26 | Fatty acids |
| 188 | Dankasterone B | C_28_H_42_O_3_ | 426.31 | Steroids |
| 189 | (22E)-9,14-Dihydroxyergosta-4,7,22-triene-3,6-dione | C_28_H_40_O_4_ | 440.29 | Steroids |
| 190 | 7-O-Succinyl macrolactin A | C_28_H_38_O_8_ | 507.26 | Macrolides |
| 191 | 11β-Acetoxyisoaustinone | C_27_H_32_O_8_ | 484.21 | Meroterpenoids |
| 192 | Austinoneol A | C_24_H_30_O_6_ | 414.20 | Meroterpenoids |
| 193 | Neoaustin | C_25_H_30_O_6_ | 426.20 | Meroterpenoids |
| 194 | Brasilianoid L | C_25_H_30_O_6_ | 426.20 | Meroterpenoids |
| 195 | Microperfuranone | C_17_H_14_O_3_ | 266.09 | Lignans |
| 196 | Butyrolactone-1 | C_24_H_24_O_7_ | 424.15 | Lignans |
| 197 | Berkeleylactone J | C_16_H_28_O_4_ | 284.20 | Macrolides |
| 198 | Aspernidine A | C_23_H_33_NO_4_ | 399.24 | Alkaloids |
| 199 | Citristerone A | C_24_H_34_O_3_ | 370.25 | Steroids |
| 200 | Citristerone B | C_25_H_34_O_3_ | 382.25 | Steroids |
| 201 | Citristerone C | C_25_H_34_O_3_ | 382.25 | Steroids |
| 202 | Citristerone D | C_25_H_34_O_3_ | 382.25 | Steroids |
| 203 | Citristerone E | C_25_H_34_O_3_ | 382.25 | Steroids |
| 204 | Amoenamide D | C_26_H_33_N_3_O_5_ | 467.24 | Alkaloids |
| 205 | Amoenucle A | C_20_H_24_N_2_O_8_ | 420.15 | Nucleosides |
| 206 | Amoenucle B | C_19_H_22_N_2_O_8_ | 406.14 | Nucleosides |
| 207 | Amoenucle C | C_25_H_27_N_3_O_10_ | 529.17 | Nucleosides |
| 208 | Amoenucle D | C_25_H_27_N_3_O_10_ | 529.17 | Nucleosides |
| 209 | Amoenucle E | C_18_H_20_N_2_O_9_ | 408.12 | Nucleosides |
| 210 | Amoenucle F | C_18_H_20_N_2_O_9_ | 408.12 | Nucleosides |
| 211 | Acylphlorostylum A | C_27_H_40_O_5_ | 444.29 | PPAPs |
| 212 | Acylphlorostylum B | C_27_H_40_O_5_ | 444.29 | PPAPs |
| 213 | Acylphlorostylum C | C_27_H_40_O_5_ | 444.29 | PPAPs |
| 214 | Acylphlorostylum D | C_27_H_40_O_5_ | 444.29 | PPAPs |
| 215 | Acylphlorostylum E | C_26_H_38_O_5_ | 430.27 | PPAPs |
| 216 | Acylphlorostylum F | C_27_H_40_O_5_ | 444.29 | PPAPs |
| 217 | Acylphlorostylum G | C_27_H_40_O_5_ | 444.29 | PPAPs |
| 218 | Longisglucinol A | C_27_H_40_O_6_ | 460.28 | PPAPs |
| 219 | Longisglucinol B | C_27_H_40_O_5_ | 444.29 | PPAPs |
| 220 | Longisglucinol C | C_27_H_40_O_5_ | 444.29 | PPAPs |
| 221 | Prenyllongnol A | C_27_H_40_O_6_ | 460.28 | PPAPs |
| 222 | Prenyllongnol B | C_27_H_40_O_6_ | 460.28 | PPAPs |
| 223 | Prenyllongnol C | C_27_H_40_O_6_ | 460.28 | PPAPs |
| 224 | Prenyllongnol D | C_27_H_40_O_6_ | 460.28 | PPAPs |
| 225 | Spihyperglucinol A | C_25_H_34_O_5_ | 414.24 | PPAPs |
| 226 | Spihyperglucinol B | C_25_H_34_O_5_ | 414.24 | PPAPs |
| 227 | Spihyperglucinol C | C_27_H_40_O_6_ | 460.28 | PPAPs |
| 228 | Spihyperglucinol D | C_27_H_40_O_6_ | 460.28 | PPAPs |
| 229 | Spihyperglucinol E | C_27_H_40_O_6_ | 460.28 | PPAPs |
| 230 | Hyperielliptone HB | C_27_H_40_O_6_ | 460.28 | PPAPs |
| 231 | Monosescinol A | C_42_H_64_O_6_ | 664.47 | PPAPs |
| 232 | Monosescinol B | C_27_H_40_O_6_ | 460.28 | PPAPs |
| 233 | Monosescinol C | C_27_H_40_O_6_ | 460.28 | PPAPs |
| 234 | Monosescinol D | C_27_H_40_O_6_ | 460.28 | PPAPs |
| 235 | Maydistacin A | C_25_H_36_O_4_ | 400.26 | Sesterterpenoids |
| 236 | Maydistacin B | C_27_H_40_O_5_ | 400.26 | Sesterterpenoids |
| 237 | Maydistacin C | C_27_H_40_O_6_ | 460.28 | Sesterterpenoids |
| 238 | Maydistacin D | C_25_H_38_O_5_ | 418.27 | Sesterterpenoids |
| 239 | Maydistacin E | C_27_H_40_O_6_ | 460.28 | Sesterterpenoids |
| 240 | Maydistacin F | C_27_H_40_O_6_ | 460.28 | Sesterterpenoids |
| 241 | Maydistacin G | C_27_H_40_O_5_ | 444.29 | Sesterterpenoids |
| 242 | Hypermonin A | C_26_H_36_O_5_ | 428.26 | PPAPs |
| 243 | Hypermonin B | C_26_H_36_O_5_ | 428.26 | PPAPs |
| 244 | Hypermonin C | C_27_H_38_O_5_ | 442.27 | PPAPs |
| 245 | Hypermonin D | C_27_H_38_O_5_ | 442.27 | PPAPs |
| 246 | Triterhyper A | C_29_H_44_O_2_ | 424.33 | Triterpenoids |
