# Supplementary material for: A novel high-throughput screen identifies phenazine-1-carboxylic acid as an inhibitor of African swine fever virus replication in primary porcine alveolar macrophages
Source: Vet Res. 2025 Feb 8;56:37. doi: 10.1186/s13567-025-01467-2 (PMC11806816; doi:10.1186/s13567-025-01467-2)
Supplement: Supplementary file 2 — Additional file 2. The primers used in this study. [file 13567_2025_1467_MOESM2_ESM.docx]

**Additional file 2 The primers used in this study.**

| Primers | Sequences (5'→3') | Descriptions |
| --- | --- | --- |
| 4L-LA-F | GGTACCCGGGAGCTCGAATTCATGAATTCTAGTATGCCTGT | For amplifying the left arm |
| 4L-LA-R | GACTTTTCTCCGGCGACCCGGGTATGAGAAAAAAAAATAATT |  |
| p72-Gluc-F | AATTATTTTTTTTTCTCATACCCGGGTCGCCGGAGAAAAGTC | For amplifying the *Gluc* gene |
| p72-Gluc-R | CATCTCTCACGAGATCGTGACTTAGTCACCACCGGCCCC |  |
| p72-EGFP-F | GGGGCCGGTGGTGACTAAGTCACGATCTCGTGAGAGATG | For amplifying the *EGFP* gene |
| p72-EGFP-R | ACCCAATATTTTTATATTTAATCCTGTGAGATCATGGCAGCT |  |
| 4L-RA-F | AGCTGCCATGATCTCACAGGATTAAATATAAAAATATTGGGT | For amplifying the right arm |
| 4L-RA-R | GTCTGCAGAAGCTTCGAATTCATTATGTAAACAGCCGCTA |  |
| D-JD-F | ATTAAACAAGGGATGCGACT | For amplifying *p72*-*Gluc* and *p72*-*EGFP* sequence |
| D-JD-R | GTTTGCTGTTACACACTCGGA |  |
