# Supplementary figures and images for: A novel high-throughput screen identifies phenazine-1-carboxylic acid as an inhibitor of African swine fever virus replication in primary porcine alveolar macrophages
Source: Vet Res. 2025 Feb 8;56:37. doi: 10.1186/s13567-025-01467-2 (PMC11806816; doi:10.1186/s13567-025-01467-2)

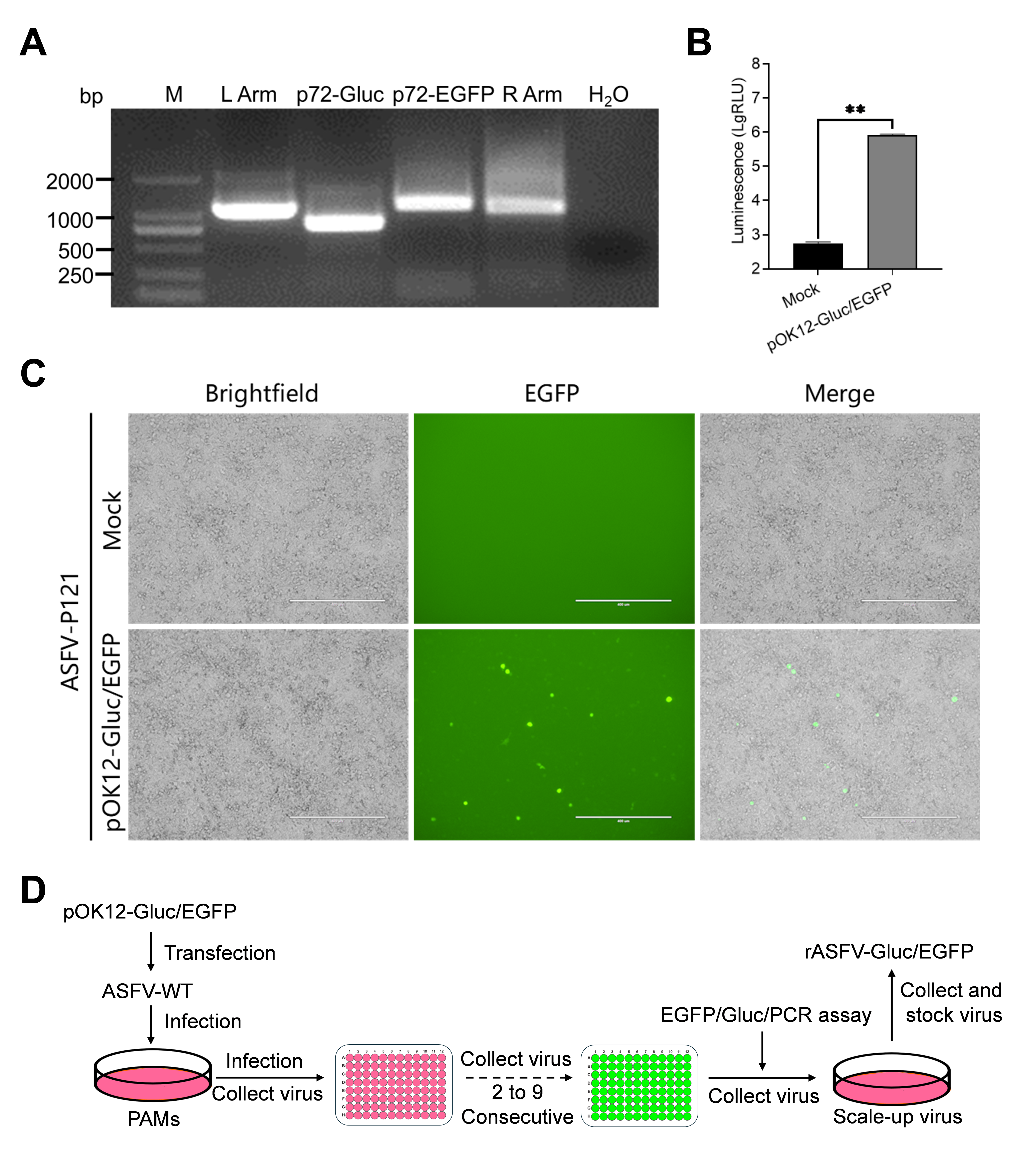

Supplement: Supplementary file 3 — Additional file 3. Generation of the transfer vector pOK12-Gluc/EGFP and the dual-reporter virus rASFV-Gluc/EGFP. (A) Generation of the transfer vector pOK12-Gluc/EGFP. The genomic fragment covering the left homology arm, p72-Gluc, p72-EGFP, and the right homology arm were amplified by PCR and analysed by agarose gel electrophoresis. (B) Gluc assay. HEK293T cells were transfected with the transfer vector pOK12-Gluc/EGFP and then infected with ASFV-P121 (MOI = 5), and the Gluc activities in the supernatants were measured at 24 hours post-infection (hpi). (C) Observation of EGFP expression. The pOK12-Gluc/EGFP transfer vector was transfected into HEK293T cells, which were inoculated with ASFV-P121 (MOI = 5), and the EGFP expression was observed by fluorescence microscopy at 24 hpi. (D) Schematic diagram of rASFV-Gluc/EGFP generation and purification. **P < 0.01. [file 13567_2025_1467_MOESM3_ESM.tif]

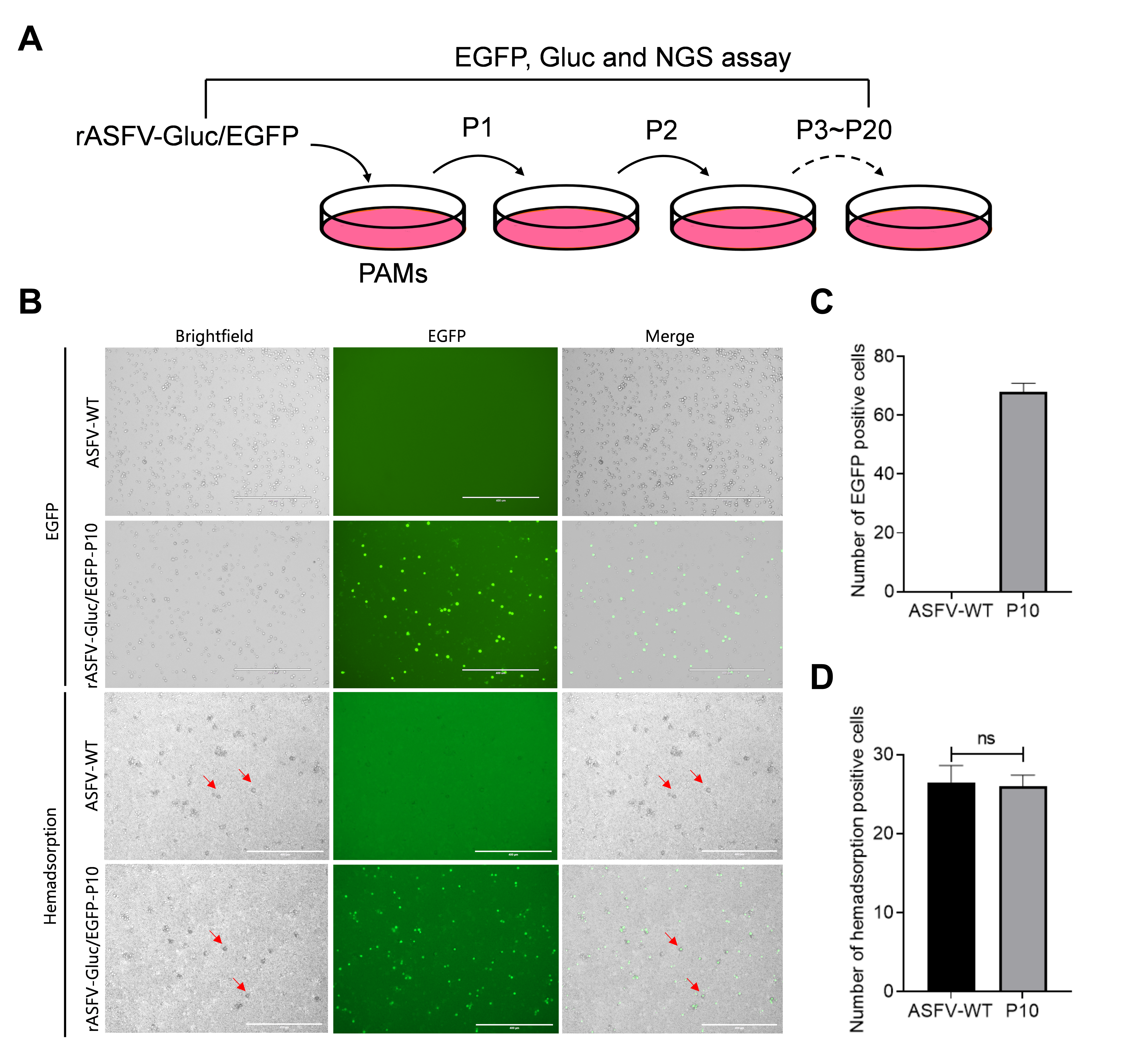

Supplement: Supplementary file 5 — Additional file 5. The genetic stability of rASFV-Gluc/EGFP. (A) Schematic diagram of continuous passaging of rASFV-Gluc/EGFP. (B to D) Hemadsorption and fluorescence assays of P10. rASFV-Gluc/EGFP was passaged in PAMs for 20 consecutive passages, with EGFP expression and hemadsorption observed at P10 by fluorescence microscopy (B). The number of EGFP-positive cells (C) or hemadsorption-positive cells (D) per field of view was measured using the ImageJ software. Red arrows indicate hemadsorption. Scale bars = 400 μm. ns, not significant. [file 13567_2025_1467_MOESM5_ESM.tif]

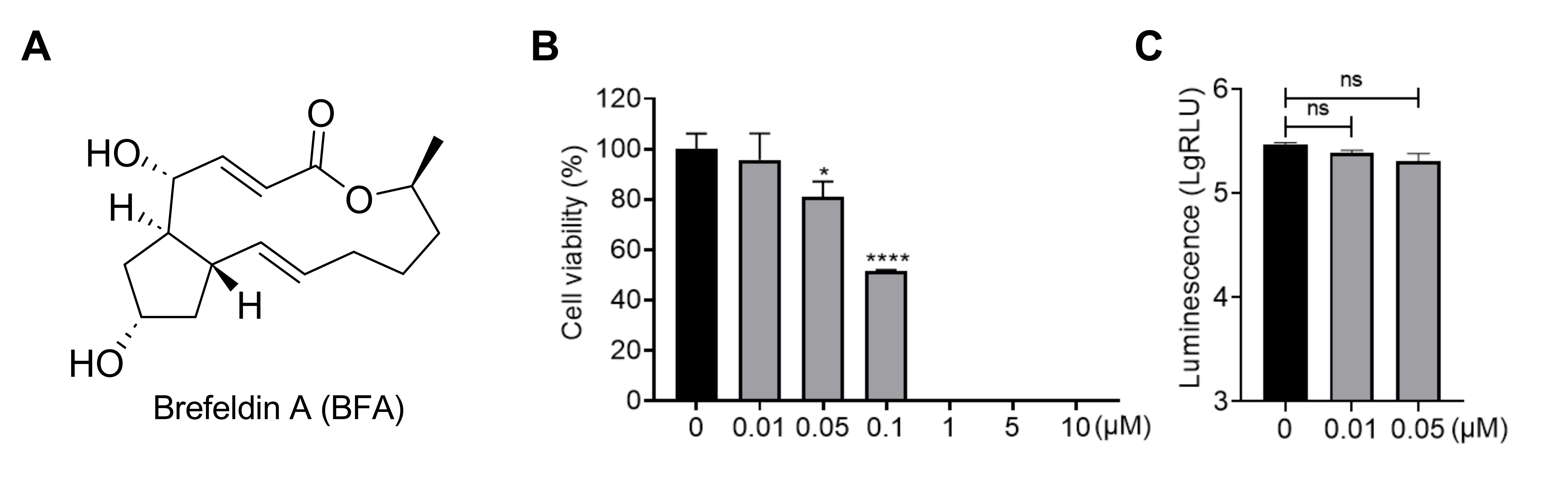

Supplement: Supplementary file 6 — Additional file 6. The cytotoxicity of brefeldin A (BFA) to PAMs and its inhibitory effects on ASFV. (A) The chemical structure of BFA. (B) The cytotoxicity of BFA to PAMs. (C) Inhibitory effects of BFA on rASFV-Gluc/EGFP. PAMs were infected with rASFV-Gluc/EGFP (MOI = 0.2) and treated with BFA (0, 0.01, or 0.05 μM), and Gluc activities were assayed at 36 hpi. *P < 0.1; ****P < 0.0001; ns, not significant. [file 13567_2025_1467_MOESM6_ESM.tif]
