# Supplementary material for: A novel high-throughput screen identifies phenazine-1-carboxylic acid as an inhibitor of African swine fever virus replication in primary porcine alveolar macrophages
Source: Vet Res. 2025 Feb 8;56:37. doi: 10.1186/s13567-025-01467-2 (PMC11806816; doi:10.1186/s13567-025-01467-2)
Supplement: Supplementary file 4 — Additional file 4. Next-generation sequencing analysis of rASFV-Gluc/EGFP. [file 13567_2025_1467_MOESM4_ESM.docx]

**Additional file 3 Next-generation sequencing analysis of rASFV-Gluc/EGFP.**

| Sample  name | Total reads | Uniquely aligned reads | Overall aligned reads | Mean coverage | Genome coverage | Nucleotide insertions | | |
| --- | --- | --- | --- | --- | --- | --- | --- | --- |
|  |  |  |  |  |  | Positions | | Length (bp) |
|  |  |  |  |  |  | Start | End |  |
| rASFV-Gluc/EGFP | 9 781 548 | 107 884 | 1.11% | 79.6% | 99.96% | 21934 | 23847 | 1894 |
| rASFV-Gluc/EGFP-P20 | 8 464 016 | 95 474 | 1.13% | 69.54% | 99.99% | 21934 | 23847 | 1894 |
